# Projecting the incidence and costs of major cardiovascular and kidney complications of type 2 diabetes with widespread SGLT2i and GLP-1 RA use: a cost-effectiveness analysis

**DOI:** 10.1007/s00125-022-05832-0

**Published:** 2022-11-21

**Authors:** Jedidiah I. Morton, Clara Marquina, Jonathan E. Shaw, Danny Liew, Kevan R. Polkinghorne, Zanfina Ademi, Dianna J. Magliano

**Affiliations:** 1 Baker Heart and Diabetes Institute, Melbourne, VIC Australia; 2 School of Public Health and Preventive Medicine, Monash University, Melbourne, VIC Australia; 3 Centre for Medicine Use and Safety, Faculty of Pharmacy and Pharmaceutical Sciences, Monash University, Melbourne, VIC Australia; 4 Adelaide Medical School, University of Adelaide, Adelaide, SA Australia; 5 Department of Nephrology, Monash Health, Clayton, VIC Australia; 6 Department of Medicine, Monash University, Melbourne, VIC Australia

**Keywords:** Cardiovascular disease, Diabetes, Glucagon-like peptide 1 receptor agonist, Health economic analysis, Kidney disease, Sodium-glucose co-transporter 2 inhibitor

## Abstract

**Aims/hypothesis:**

Whether sodium–glucose co-transporter 2 inhibitors (SGLT2is) or glucagon-like peptide-1 receptor agonists (GLP-1 RAs) are cost-effective based solely on their cardiovascular and kidney benefits is unknown. We projected the health and economic outcomes due to myocardial infarction (MI), stroke, heart failure (HF) and end-stage kidney disease (ESKD) among people with type 2 diabetes, with and without CVD, under scenarios of widespread use of these drugs.

**Methods:**

We designed a microsimulation model using real-world data that captured CVD and ESKD morbidity and mortality from 2020 to 2040. The populations and transition probabilities were derived by linking the Australian Diabetes Registry (1.1 million people with type 2 diabetes) to hospital admissions databases, the National Death Index and the ESKD Registry using data from 2010 to 2019. We modelled four interventions: increase in use of SGLT2is or GLP-1 RAs to 75% of the total population with type 2 diabetes, and increase in use of SGLT2is or GLP-1 RAs to 75% of the secondary prevention population (i.e. people with type 2 diabetes and prior CVD). All interventions were compared with current use of SGLT2is (20% of the total population) and GLP-1 RAs (5% of the total population). Outcomes of interest included quality-adjusted life years (QALYs), total costs (from the Australian public healthcare perspective) and the incremental cost-effectiveness ratio (ICER). We applied 5% annual discounting for health economic outcomes. The willingness-to-pay threshold was set at AU$28,000 per QALY gained.

**Results:**

The numbers of QALYs gained from 2020 to 2040 with increased SGLT2i and GLP-1 RA use in the total population (*n*=1.1 million in 2020; *n*=1.5 million in 2040) were 176,446 and 200,932, respectively, compared with current use. Net cost differences were AU$4.2 billion for SGLT2is and AU$20.2 billion for GLP-1 RAs, and the ICERs were AU$23,717 and AU$100,705 per QALY gained, respectively. In the secondary prevention population, the ICERs were AU$8878 for SGLT2is and AU$79,742 for GLP-1 RAs.

**Conclusions/interpretation:**

At current prices, use of SGLT2is, but not GLP-1 RAs, would be cost-effective when considering only their cardiovascular and kidney disease benefits for people with type 2 diabetes.

**Graphical abstract:**

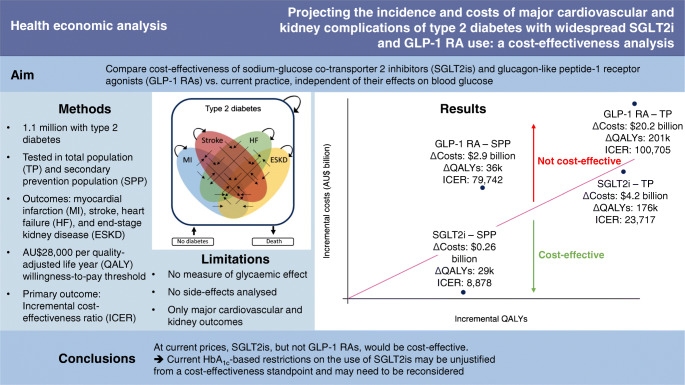

**Supplementary Information:**

The online version contains peer-reviewed but unedited supplementary material available at 10.1007/s00125-022-05832-0.

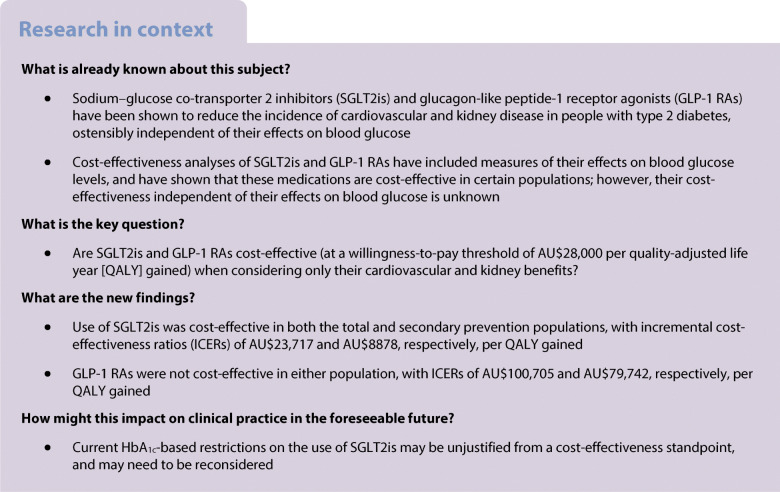



## Introduction

Sodium–glucose co-transporter 2 inhibitors (SGLT2is) and glucagon-like peptide-1 receptor agonists (GLP-1 RAs) have been shown to reduce the incidence of cardiovascular and kidney disease in people with type 2 diabetes, ostensibly independent of their effects on blood glucose [1, 2]. For example, SGLT2i use leads to a 33% reduction (95% CI 26, 38) in hospitalisation for heart failure (HF) and a 35% reduction (95% CI 19, 47) in the incidence of end-stage kidney disease (ESKD) [3, 4], and GLP-1 RA use leads to a 10% reduction (95% CI 2, 17) in myocardial infarction (MI) and a 17% reduction (95% CI 8, 24) reduction in stroke [2]. Moreover, SGLT2is have been shown to reduce the incidence of cardiovascular and kidney disease in people with and without diabetes [5, 6], suggesting that these medications should be considered for prevention of cardiovascular and kidney disease, irrespective of HbA_1c_ levels. Indeed, guidelines for people with type 2 diabetes were updated in 2019 to recommend their use in people with, or at risk for, cardiovascular and kidney disease [7].

Despite these benefits and changes to management guidelines, uptake of SGLT2is and especially GLP-1 RAs among people with type 2 diabetes has been limited [8, 9]. An important barrier to uptake is the high cost of these medications [10]. Payers (including governments) base their decisions on whether and to whom a medication will be made available based on the cost-effectiveness of that medication. However, even government payers still restrict use of SGLT2is and GLP-1 RAs to those for whom at least one other medication has failed to achieve adequate glycaemic control [11, 12].

Importantly, payer reimbursement restrictions are probably in place because previous cost-effectiveness analyses of these medications have considered at least some measure of their glycaemic benefits [13, 14], while whether SGLT2is and GLP-1 RAs are cost-effective solely on the basis of their benefits on cardiovascular and kidney disease has never been studied as far as we are aware. This information is essential to encourage payers to expand access to these medications earlier in the course of diabetes and irrespective of HbA_1c_ levels.

Therefore, using a large, real-world population with type 2 diabetes, we constructed a model to assess the cost-effectiveness of widespread use of SGLT2is and GLP-1 RAs in people with type 2 diabetes, considering only major cardiovascular and kidney outcomes.

## Methods

### Model overview

**Fig. 1:**
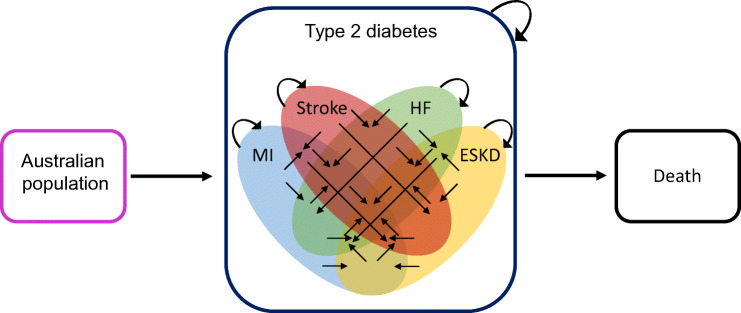
Schematic of the model. The model begins on 1 January 2020 with the entire population with type 2 diabetes in Australia. Each cycle (1 year), people with type 2 diabetes are at risk for MI, stroke, HF, ESKD and death, with the number experiencing each event in the cycle tracked. If an individual experiences an event they have not had before, they then transition to the relevant health state (straight black arrows). If an individual does not experience an event, or experiences only the event of the type they have previously had, they remain in the same health state for the next cycle (circular arrows). There are 17 possible health states, represented as either absence of all of MI, stroke, HF and ESKD (i.e. people with type 2 diabetes not contained within the Venn diagram), any combination of these four outcomes (the 15 spaces within the Venn diagram), or death. Additionally, at each cycle, a population with incident type 2 diabetes is added, who enter the model in their respective health states

We designed a microsimulation model using real-world, individual-level data that captured the incidence and costs of ESKD, non-fatal hospitalisations for MI, stroke and HF, and all-cause mortality among people with type 2 diabetes in Australia from 2020 to 2040 (Fig. 1). The model began with the entire Australian population with type 2 diabetes in 2020. Baseline health states were assigned based on having had a hospitalisation for MI, stroke or HF from 2010 to 2019, or having developed ESKD at any point before 2019. The cohort was then aged in yearly cycles, experiencing MI, stroke, HF and ESKD events, and transitioning between health states. Thus, for each cycle, people with type 2 diabetes are at risk for MI, stroke, HF, ESKD and death, with the number experiencing each event in the cycle being tracked. If an individual has an event they have not had before, they then transition to the relevant health state reflecting all prior conditions they have experienced (a total of 16 possible alive health states or death). If an individual does not experience an event, or experiences only an event of a type they have previously had, they remain in the same health state for the next cycle.

The incidence of each event and transition probabilities between health states were modelled via Poisson regression, based on current age, diabetes duration, age at diagnosis of diabetes, sex and health state (see electronic supplementary material [ESM] Methods). Rates were validated against existing data (ESM Methods—validation of transition probabilities and model structure; ESM Figs 4–9). Additionally, each year, a cohort with new-onset type 2 diabetes is added (ESM Methods—diabetes incidence). The outcomes captured were incident cases of ESKD, hospitalisation for MI, stroke and HF, years of life lived, quality-adjusted life years (QALYs), healthcare costs and societal costs. The primary outcome was the incremental cost-effectiveness ratio (ICER), defined as cost per QALY gained, with the willingness-to-pay threshold set at AU$28,000 per QALY [15]. We evaluated outcomes in two populations: the total population with type 2 diabetes, and the secondary prevention population, which comprised everyone with type 2 diabetes and prior CVD. Prior CVD was defined as having had an admission (either an actual admission from 2010 to 2019, or a modelled admission from 2020 to 2040) for an MI, stroke or HF. We adopted both a healthcare and societal perspective, with 5% annual discounting for QALYs and costs as per Australian guidelines [16].

### Interventions

We modelled four interventions: increase in use of SGLT2is or GLP-1 RAs to 75% of the total population with type 2 diabetes, and increase in use of SGLT2is or GLP-1 RAs to 75% of the secondary prevention population. All interventions were compared with the use of SGLT2is and GLP-1 RAs in 2019 (the latest year with available data), i.e. approximately 20% of the population with type 2 diabetes for SGLT2is and approximately 5% for GLP-1 RAs [9]. Use of these medications in 2019 did not vary substantially for people with and without prior CVD. People with ESKD were assumed to discontinue both medications. Estimates of the effects of SGLT2is and GLP-1 RAs on cardiovascular and kidney disease and all-cause mortality were assumed to be class effects and were thus drawn from the recent meta-analyses of major-outcomes trials [2–4]; the total population sizes in the meta-analyses ranged from 38,723 to 79,799 with median follow-up durations for the included trials ranging from 0.8 to 5.4 years. HRs and 95% CIs used to model the effects of each medication are presented in Table 1. We assumed that the effects of the medications were independent of each other [17] and maintained for the entire duration of use. Effects of SGLT2is on ESKD were modelled with a 2-year delay, as previously described [18]. Briefly, this was done to reflect the expected lack of an immediate benefit on ESKD, as most individuals who develop ESKD have an eGFR<30 ml/min per 1.73m^2^ in the 2 years preceding ESKD [19], and are thus not eligible for SGLT2is.

**Table 1:** Key model inputs

Input	Value	Distribution	Source
Population			
Diabetes prevalence at baseline	Sex, age and duration of diabetes-specific prevalence	Fixed	NDSS linkage, multiplied to encompass whole Australian population
Health states at baseline	Sex, age and duration of diabetes-specific prevalence	Fixed	NDSS linkage
Transition probabilities			
Diabetes incidence	Sex and age-specific incidence	See ESM Fig. 1	NDSS linkage/Australian population estimates and projections (see ESM)
Transitions between health states	Sex, age and duration of diabetes-specific rates	See ESM Figs 2 and 3	NDSS linkage (see ESM)
Hazard ratios for SGLT2is			
MI	0.91 (0.84; 0.99)	Log-normal	[3]
Stroke	0.98 (0.88; 1.09)	Log-normal	[3]
Hospitalisation for HF	0.67 (0.62; 0.74)	Log-normal	[3]
ESKD	0.65 (0.53; 0.81)	Log-normal	[4]
All-cause mortality	0.88 (0.83; 0.94)	Log-normal	[3]
Hazard ratios for GLP-1 RAs			
MI	0.90 (0.83; 0.98)	Log-normal	[2]
Stroke	0.83 (0.76; 0.92)	Log-normal	[2]
Hospitalisation for HF	0.89 (0.82; 0.98)	Log-normal	[2]
ESKD	Base case: no effect	Log-normal	–
All-cause mortality	0.88 (0.82; 0.94)	Log-normal	[2]
Acute costs (AU$)			
MI	13,198 (±25%)	Gamma	NDSS linkage/DRG codes
Stroke	14,318 (±25%)	Gamma	NDSS linkage/DRG codes
Hospitalisation for HF	10,488 (±25%)	Gamma	NDSS linkage/DRG codes
Death	8795 (±25%)	Gamma	NDSS linkage/DRG codes
Chronic costs (AU$, annual)			
SGLT2is	724	Fixed	NDSS linkage/PBS
GLP-1 RAs	1709	Fixed	NDSS linkage/PBS
Diabetes (no complications)	3281 (2575; 3986)	Gamma	[25]
Diabetes with prior CVD	8110 (6221; 10,000)	Gamma	[25]
Diabetes with ESKD	120,000 (±25%)	Gamma	Assumption based on [26]
Utilities^a^			
Diabetes without complication	0.785 (0.681; 0.889)	Beta	[22]
Chronic disutility for MI	−0.055 (−0.067; −0.042)	Beta	[22]
Chronic disutility for stroke	−0.164 (−0.222; −0.105)	Beta	[22]
Chronic disutility for HF	−0.108 (−0.169; −0.048)	Beta	[22]
Chronic disutility for ESKD	−0.164 (−0.274; −0.054)^b^	Beta	[22]
Acute disutility for MI	−0.03 (±25%)	Beta	[24]
Acute disutility for stroke	−0.05 (±25%)	Beta	[24]
Acute disutility for HF hospitalisation	−0.03 (±25%)	Beta	[24]
Indirect costs			
Employment and participation	Internal to model: diabetes, age and sex-specific		Workforce participation and unemployment [29], mean earnings [28] and effect of diabetes on workforce participation [35]
Not being in the workforce prevalence ratio			
For MI	1.46		[36]
For stroke	1.92		[36]
For HF	1.83		[36]
Workforce participation among people with ESKD	50%		Assumption based on [37, 38]
Mean sick leave (days)			
For acute MI	60		[30]
For acute stroke	90		[31]
For HF hospitalisation	5		Assumption
Absenteeism (days/year)			
For diabetes	3.0		[32]
For MI	5.5		[33]
For stroke	5.5		[33]
For HF	5.5		[33]
For ESKD	6.0		[34]

All costs were adjusted to 2020 AU$ using the Health Price Index [39]

^a^Adjusted for age within model

^b^Haemodialysis only, being conservative

DRG, diagnosis-related group; PBS, Australian Pharmaceutical Benefits Scheme

### Model population and transition probabilities

The population and data sources from which transition rates for the model were derived have been described previously [18, 20]. Briefly, the National Diabetes Services Scheme (NDSS) includes 80–90% of people with diagnosed diabetes in Australia. To estimate transition probabilities for this study, we included all Australians with type 2 diabetes who do not identify as Aboriginal or Torres Strait Islander who were registered on the NDSS in four Australian states (80% of the NDSS) at any point between 1 July 2010 and 30 June 2019 (median age 68.9 [IQR 59.0–77.6]; 55% male; ESM Table 1). This cohort was linked to the Australia and New Zealand Dialysis and Transplant Registry (ANZDATA), the National Death Index, and hospital-admitted patient data collections. ANZDATA is a complete registry of all people who receive kidney replacement therapy, and was used to model the incidence of ESKD. The National Death Index records all deaths that occur in Australia, and was used to model all-cause mortality. Hospital-admitted datasets record all admissions to public hospitals, and were used to model the incidence of non-fatal hospitalisations for MI, stroke and HF [20]. A detailed description of the methods used to derive transition probabilities is provided in ESM Methods.

### Utilities

Utilities are used to quantify the perception of health for an individual’s health state, and range from 0 (death) to 1 (perfect health). All utility values used in this study were derived via the EuroQol-5 dimensions questionnaire [21]. The utility for each health state in this study was as recommended in a review of utility values for type 2 diabetes and its complications [22]. These values were adjusted for age to reflect the change in quality of life with age [23]. To be conservative, we used the maximum possible disutility for each health state.

We also applied acute disutilities for each MI, stroke and HF event (Table 1). These were 0.12 for MI, 0.21 for stroke, and 0.11 for HF [24]. During the cycle that an event occurred in, these acute disutilities were applied for 3 months of the cycle. Events (MI, stroke, HF, ESKD and death) were assumed to occur at the mid-point of the cycle; thus, during a cycle in which an event occurred, the utility for the initial health state was applied for 6 months, followed by the utility of the final health state for 6 months. All (dis)utilities are shown in Table 1.

### Costs

For healthcare costs, chronic costs of each health state were derived from those published in Lee et al [25], with the exception of ESKD, for which costs were based on a recent Australian costing study of ESKD [26]. Acute hospitalisation costs were derived from the NDSS-linked dataset described above. For treatment costs, we assumed full adherence to medications, and derived medication costs directly from the Australian Pharmaceutical Benefits Scheme (https://www.pbs.gov.au/pbs/home). The most common SGLT2i in Australia in 2020 was empagliflozin; thus, the cost of SGLT2is was based on the cost of empagliflozin in June 2020 and was set at $724 per year. Similarly, dulaglutide was the most common GLP-1 RA in 2020, with an annual cost of $1709.

Societal costs were estimated using the human capital approach [27]. We included costs of lost earnings due to absenteeism (acute and chronic), workforce dropout due to CVD or ESKD, and loss of future earnings from premature mortality (death before retirement age, which was set at 67 years). All indirect costs were calculated by multiplying lost work time from the current age until age 67 or the year 2040 (whichever came first) by the sex-specific mean earnings in Australia in May 2020 (AU$80,235 for men and $AU56,494 for women [28]), adjusted for age and sex-specific workforce participation and unemployment rates in December 2019 [29]. Acute absenteeism periods for MI and stroke were set at 60 and 90 days, respectively [30, 31]. We conservatively assumed that a hospitalisation for HF would lead to 5 days of sick leave. Chronic absenteeism for diabetes was set at 3.0 days/year [32], that for CVD was set at 5.5 days/year [33], and that for ESKD was set at 6.0 days/year [34]. The effect of diabetes on workforce participation was drawn from a study of Australian National Health Surveys [35]. For CVD health states, workforce non-participation was calculated by multiplying the workforce non-participation rate by the prevalence ratio of non-participation from a large Australian cross-sectional study (1.46 for MI, 1.92 for stroke and 1.83 for HF [36]). We assumed that 50% of people of working age with ESKD were employed [37, 38]. Healthcare and societal cost inputs are shown in Table 1. All costs were adjusted to 2020 AU$ using the Health Price Index [39].

### Scenario analyses

We performed a number of scenario analyses. (1) Because people with type 2 diabetes who start using an SGLT2i or GLP-1 RA will probably delay initiation of another glucose-lowering medication, costs will be saved. Therefore, we performed a scenario analysis in which we assumed that everyone who started an SGLT2i or GLP-1 RA would have initiated a different glucose-lowering medication at the same time, and thus the cost of SGLT2is and GLP-1 RAs was reduced by the mean cost of all other glucose-lowering medications ($288; calculated from the NDSS). (2) We assessed use of SGLT2is in combination with metformin, which reduces the annual cost of SGLT2is to $552. (3) As patents for SGLT2is and GLP-1 RAs will expire before 2040, we performed a scenario analysis in which the cost of each was reduced by 50% to simulate the reduction in price [40]. (4) We modelled adherence to SGLT2is and GLP-1 RAs as 75% and 82%, respectively; these are the lowest values from cardiovascular outcomes trials of the more common SGLT2is and GLP-1 RAs used in Australia [41, 42]. Because efficacy estimates were derived from intention-to-treat analyses, which account for non-adherence, we only applied the reduction in adherence to the price of the medications, not their efficacy. (5) An analysis was performed using the approximate mean annual cost of SGLT2is and GLP-1 RAs from the USA (conservatively: AU$7000 for SGLT2is and AU$10,500 for GLP-1 RAs). (6,7) Analyses were performed in which the incidence of type 2 diabetes was decreased or increased at 4% per year [43]. (8) The base-case scenario assumed constant mortality rates; we also performed a scenario analysis in which mortality declined at a rate of 2.2% per year for men and 1.3% per year for women [18]. (9) While it has never been shown in a dedicated trial, a recent network meta-analysis [44] suggested that GLP-1 RAs may reduce the risk for ESKD (HR 0.78; 95% CI 0.67, 0.92); thus, we included this effect in a scenario analysis. (10,11) Analyses were performed in which use of each medication increased to 50% or 100%. (12) In the base-case scenario, transition probabilities were estimated using public hospital data only [20]; thus, in this scenario analysis, we projected outcomes including private hospital data. (13) An analysis was performed in which the timeframe was altered to 2020−2030. (14) Because all trial data informing this analysis were obtained from relatively short-term trials, we performed a scenario analysis in which the efficacy of each medication on all outcomes decreased by 5% per year from 2020 to 2040. (15–17) Analyses were performed in which the discounting rate was 0, 3 or 6%.

Finally, we also performed a threshold analysis to determine the cost at which GLP-1 RAs would become cost-effective using a step size of AU$50 per annum.

### Sensitivity analyses

To quantify the effects of uncertainty in the input variables on the results, we performed one-way sensitivity analyses using the lower and upper bounds outlined in Table 1. To estimate the combined uncertainty in outcomes, we performed probabilistic sensitivity analyses using 1000 Monte Carlo simulations based on the uncertainty in the model variables, drawing model variables randomly from the distributions in Table 1. The uncertainty intervals represent the 2.5th and 97.5th centile values from these simulations. Statistical analyses were performed using Stata statistical software, version 16 (StataCorp, USA).

## Results

### Base-case results

The prevalence of diabetes was projected to grow from 1.13 million in 2020 to 1.45 million in 2040 under the base-case scenario. Compared with current use (20%) of SGLT2is, widespread use (75%) in the total population with type 2 diabetes was projected to prevent 13,376 non-fatal MIs, 117,240 HF hospitalisations, 6871 ESKD events and 35,989 deaths, but increase the number of non-fatal strokes by 1363 (Table 2). Widespread use (75%) of GLP-1 RAs in the total population with type 2 diabetes was projected to prevent 16,455 MIs, 20,409 strokes, 37,100 HF hospitalisations and 39,917 deaths, but increase the number of ESKD events by 170. These strategies resulted in a gain of 400,018 years of life lived and 176,446 QALYS for SGLT2is, and a gain of 460,028 years of life lived and 200,932 QALYs for GLP-1 RAs. From the Australian public healthcare perspective, this came at an incremental cost of AU$4.2 billion for SGLT2is and AU$20.2 billion for GLP-1 RAs, with corresponding ICERs of AU$23,717 and AU$100,705 per QALY gained, respectively. ICERs from a societal perspective were AU$17,082 and AU$94,463 per QALY gained, respectively (Table 2).

**Table 2:** Results from the base-case analysis

	Current use	SGLT2i use		GLP-1 RA use	
		Absolute value	Difference to current use	Absolute value	Difference to current use
Total population					
MI	294,424 (260,175; 330,657)	281,048 (243,480; 321,305)	−13,376 [−4.5] (−27,637; 2205)	277,969 (241,649; 316,661)	−16,455 [−5.6] (−35,690; 1875)
Stroke	187,474 (162,632; 217,116)	188,838 (161,356; 224,032)	1363 [0.7] −(11,444; 15,159)	167,065 (142,883; 196,575)	−20,409 [−10.9] (−32,409; −7084)
HF	492,696 (439,445; 550,194)	375,456 (329,505; 432,308)	−117,240 [−23.8] −(144,923; −90,501)	455,597 (394,399; 531,478)	−37,100 [−7.5] (−72,766; 8408)
ESKD	34,819 (27,410; 44,958)	27,947 (21,421; 36,902)	−6871 [−19.7] (−9881; −3662)	34,989 (27,561; 44,927)	170 [0.5] (−218; 582)
Death	1,055,719 (1,031,712; 1,076,349)	1,019,730 (990,640; 1,047,067)	−35,989 [−3.4] (−50,044; −21,660)	1,015,802 (985,267; 1,044,791)	−39,917 [−3.8] (−58,329; −21,711)
YLL	26,858,603 (26,517,388; 27,190,840)	27,258,621 (26,882,652; 27,645,008)	400,018 [1.5] (228,067; 563,317)	27,318,630 (26,903,992; 27,704,272)	460,028 [1.7] (246,692; 676,693)
QALYs	13,041,414 (11,168,379; 14,734,493)	13,217,859 (11,328,445; 14,958,538)	176,446 [1.4] (106,318; 251,600)	13,242,345 (11,359,254; 14,960,392)	200,932 [1.5] (114,336; 292,988)
Acute healthcare costs (AU$)	12,762,181,792	11,716,415,616	−1,045,766,176	11,966,683,424	−795,498,368
Chronic healthcare costs (AU$)	84,610,329,344	83,047,491,072	−1,562,838,272	85,315,993,856	705,664,512
SGLT2i costs (AU$)	2,416,672,032	9,189,991,296	6,773,319,264	2,453,092,328	36,420,296
GLP-1 RA costs (AU$)	1,426,136,920	1,446,196,612	20,059,692	21,714,441,280	20,288,304,360
Total healthcare costs (AU$)	101,215,320,088 (89,376,899,072; 114,824,241,152)	105,400,094,596 (93,676,306,432; 118,355,304,448)	4,184,774,508 [4.1] (2,897,340,928; 5,460,916,736)	121,450,210,888 (109,713,637,376; 135,190,126,592)	20,234,890,800 [20.0] (19,255,119,872; 21,226,565,632)
Acute absenteeism costs (AU$)	577,454,074	552,360,488	−25,093,586	525,483,998	−51,970,076
Chronic absenteeism costs (AU$)	4,218,623,992	4,222,893,240	4,269,248	4,226,116,736	7,492,744
Non-participation costs – morbidity (AU$)	39,223,259,392	39,025,889,280	−197,370,112	39,117,028,864	−106,230,528
Non-participation costs – mortality (AU$)	13,720,862,280	12,768,310,072	−952,552,208	12,617,259,608	−1,103,602,672
Total productivity costs (AU$)	57,740,199,738	56,569,453,080	−1,170,746,658	56,485,889,206	−1,254,310,532
Total societal costs (AU$)	158,955,519,826	161,969,547,676	3,014,027,850	177,936,100,094	18,980,580,268
ICER – YLL	–	–	10,461 (7,112; 17,364)	–	43,986 (31,023; 79,669)
ICER – QALYs	–	–	23,717 (15,600; 38,230)	–	100,705 (70,648; 172,369)
SICER – YLL	–	–	7535	–	41,260
SICER – QALYs	–	–	17,082	–	94,463
Secondary prevention population					
MI	146,717 (125,436; 170,861)	141,315 (119,027; 167,224)	−5402 [−3.7] (−11,855; 1403)	140,506 (118,429; 165,096)	−6211 [−4.2] (−14,960; 1840)
Stroke	80,074 (66,412; 96,582)	81,069 (66,253; 99,417)	996 [1.2] −(4088; 6551)	72,607 (59,652; 88,859)	−7467 [−9.3] (−12,516; −1937)
HF	340,999 (298,456; 388,885)	274,509 (236,224; 319,651)	−66,490 [−19.5] (−84,414; −50,131)	322,762 (278,285; 377,927)	−18,236 [−5.3] (−40,349; 8250)
ESKD	13,215 (10,166; 17,494)	10,891 (8,169; 14,775)	−2324 [−17.6] (−3497; −1040)	13,529 (10,390; 17,688)	314 [2.4] (140; 519)
Death	345,761 (335,338; 355,790)	338,761 (327,976; 349,711)	−7000 [−2.0] (−10,274; −3680)	337,504 (326,232; 348,411)	−8257 [−2.4] (−12,681; −4058)
YLL	3,717,454 (3,590,150; 3,863,870)	3,795,711 (3,654,660; 3,949,358)	78,256 [2.1] (38,004; 116,395)	3,814,481 (3,675,913; 3,966,151)	97,027 [2.6] (46,949; 148,382)
QALYs	1,502,744 (1,242,415; 1,740,208)	1,532,101 (1,269,201; 1,776,744)	29,357 [2.0] (14,365; 44,161)	1,538,834 (1,275,072; 1,781,656)	36,090 [2.4] (17,759; 55,934)
Acute healthcare costs (AU$)	5,725,633,728	5,234,765,088	−490,868,640	5,442,878,736	−282,754,992
Chronic healthcare costs (AU$)	23,697,541,184	23,552,370,368	−145,170,816	24,177,167,296	479,626,112
SGLT2i costs (AU$)	315,470,895	1,208,192,892	892,721,997	323,048,483	7,577,588
GLP-1 RA costs (AU$)	186,167,044	190,129,064	3,962,021	2,859,581,440	2,673,414,397
Total healthcare costs (AU$)	29,924,812,851 (25,421,770,752; 34,592,534,528)	30,185,457,412 (25,637,419,008; 34,962,751,488)	260,644,562 [0.9] (−152,687,200; 671,996,224)	32,802,675,955 (28,157,343,744; 37,616,013,312)	2,877,863,105 [9.6] (2,486,327,296; 3,271,812,608)
Acute absenteeism costs (AU$)	161,058,816	153,736,072	−7,322,744	148,796,117	−12,262,699
Chronic absenteeism costs (AU$)	489,154,224	491,669,818	2,515,594	491,845,918	2,691,694
Non-participation costs – morbidity (AU$)	9,126,613,760	9,158,692,192	32,078,432	9,173,791,968	47,178,208
Non-participation costs – mortality (AU$)	2,408,758,659	2,262,304,116	−146,454,543	2,243,937,860	−164,820,799
Total productivity costs (AU$)	12,185,585,459	12,066,402,198	−119,183,261	12,058,371,863	−127,213,596
Total societal costs (AU$)	42,110,398,310	42,251,859,610	141,461,301	44,861,047,818	2,750,649,509
ICER – YLL	–	–	3331 (−2368; 7636)	–	29,660 (21,424; 54,609)
ICER – QALYs	–	–	8878 (−6046; 20,991)	–	79,742 (56,489; 143,995)
SICER – YLL	–	–	1808	–	28,349
SICER – QALYs	–	–	4819	–	76,217

Differences to current use are presented as absolute difference [% difference] (95% CI)

All costs are presented in 2020 AU$. All health economic outcomes have been subject to 5% annual discounting

YLL, years of life lived; SICER, incremental cost-effectiveness ratio from a societal perspective

Among the secondary prevention population (*n*=95,247 at baseline), widespread SGLT2i use was projected to prevent 5402 non-fatal MIs, 66,490 HF hospitalisations, 2324 ESKD events and 7000 deaths, but increase the number of non-fatal strokes by 996. Widespread use of GLP-1 RAs was projected to prevent 6211 MIs, 7467 strokes, 18,236 HF hospitalisations and 8257 deaths, but increase the number of ESKD events by 314. The incremental number of QALYs associated with SGLT2i use was projected at 29,357, and widespread use of SGLT2is was projected to lead to an incremental healthcare cost of AU$0.3 billion, resulting in an ICER of AU$8878 per QALY gained. For GLP-1 RAs, projected QALY gain was 36,090 at a total healthcare cost increment of AU$2.9 billion, resulting in an ICER of AU$79,742 per QALY gained. From a societal perspective, the ICERs for SGLT2is and GLP-1 RAs were AU$4819 and AU$76,217 per QALY gained, respectively (Table 2).

### Subgroup analysis

The results from subgroup analyses are shown in ESM Tables 2–7. For the total population scenarios, men were projected to see a greater benefit from both SGLT2is and GLP-1 RAs than women, resulting in lower ICERs for men (the ICERs for SGLT2is were AU$20,361 per QALY for men and AU$28,377 per QALY for women, and those for GLP-1 RAs were AU$96,021 per QALY for men and AU$107,169 per QALY for women). Results were more similar for men and women in the secondary prevention population.

Considering only events within various age groups, the ICERs indicated that SGLT2is were not cost-effective for people aged 10–39 years or 40–59 years for any intervention (although the ICER for SGLT2is in the secondary prevention population was 29,420), while SGLT2is were cost-effective among people aged 60–79 years (ICERs of 28,002 and 328 in the primary prevention and secondary prevention populations, respectively) and ≥80 years, but GLP-1 RAs were not.

### Scenario analyses

**Fig. 2:**
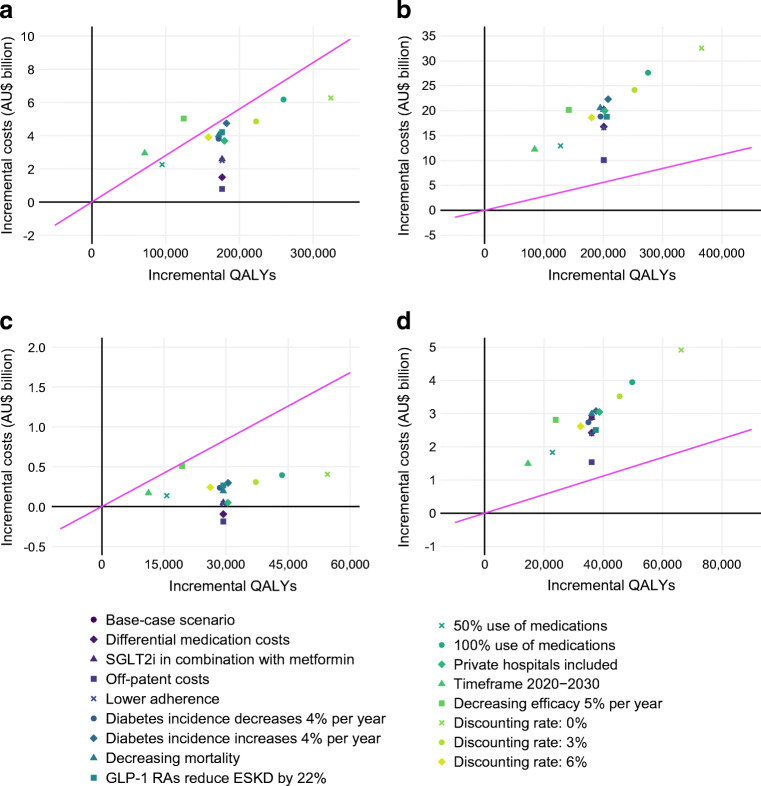
Results from scenario analyses in common cost-effectiveness planes. Incremental costs and benefits are as compared with the current use under each scenario. Not shown are results from the US medication price scenario, as the costs were too large to fit in a common plane (all results are shown in ESM Table 8). The pink line represents the AU$28,000 per QALY willingness-to-pay threshold. All costs are in 2020 Australian dollars. All health economic outcomes have been subject to 5% annual discounting unless otherwise indicated. (**a**) SGLT2i/total population, (**b**) GLP-1 RA/total population, (**c**) SGLT2i/secondary prevention population, (**d**) GLP-1 RA/secondary prevention population

The results from scenario analyses are shown in Fig. 2. SGLT2is remained cost-effective at a threshold of AU$28,000 per QALY gained under all scenarios in the total and secondary prevention populations, except when the price of SGLT2is was set at AU$7000 (US price), when the timeframe was reduced to 2020–2030, and when the efficacy was reduced by 5% per year. In the differential medication cost and off-patent cost scenarios, increased SGLT2i use was dominant over current use in the secondary prevention population. GLP-1 RAs were not cost-effective at AU$28,000 per QALY under any scenario. The results of the threshold analysis revealed that, under the base-case assumptions, GLP-1 RAs would be cost-effective at an annual cost of AU$450 in the total population and AU$500 in the secondary prevention population.

### Sensitivity analyses

**Fig. 3:**
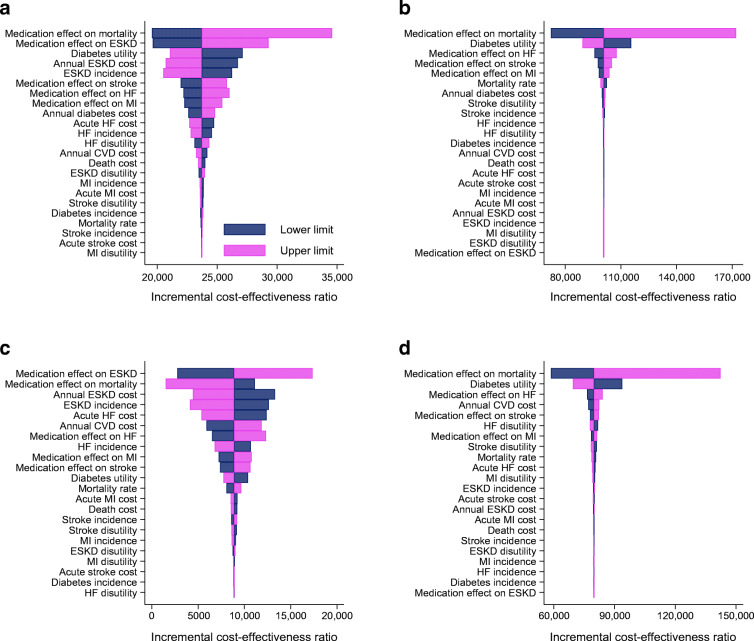
Tornado diagrams showing results from one-way sensitivity analyses. Tornado diagrams display the uncertainty in the model primary outcome (ICER) associated with variation in the input variables. Lower-limit ICERs are the ICERs under the lower limit of uncertainty associated with the variable, and upper-limit ICERs are the ICERs under the upper limit of uncertainty. For example, the effect of GLP-1 RAs on all-cause mortality is estimated using the HR of 0.88 (95% CI 0.82, 0.94); thus, the lower limit for this variable is 0.82 and the upper limit is 0.94, and the ICERs displayed in the tornado diagram are the ICERs when the model is run using these values, with the middle value representing the ICER using the point estimate of 0.88. All health economic outcomes have been subject to 5% annual discounting. (**a**) SGLT2i/total population, (**b**) GLP-1 RA/total population, (**c**) SGLT2i/secondary prevention population, (**d**) GLP-1/RA secondary prevention population

Figure 3 shows the results of the one-way sensitivity analyses. SGLT2i models were most sensitive to the effect of SGLT2is on all-cause mortality and ESKD, as well as the underlying incidence of ESKD in the model and annual cost of ESKD. Models of GLP-1 RAs were most sensitive to the effects of GLP-1 RAs on all-cause mortality, with the ICERs varying from AU$72,318 to AU$172,072 per QALY in the total population and AU$58,588 to AU$142,317 in the secondary prevention population for the lower and upper bounds of the 95% CI of the HR for GLP-1 RAs.

**Fig. 4:**
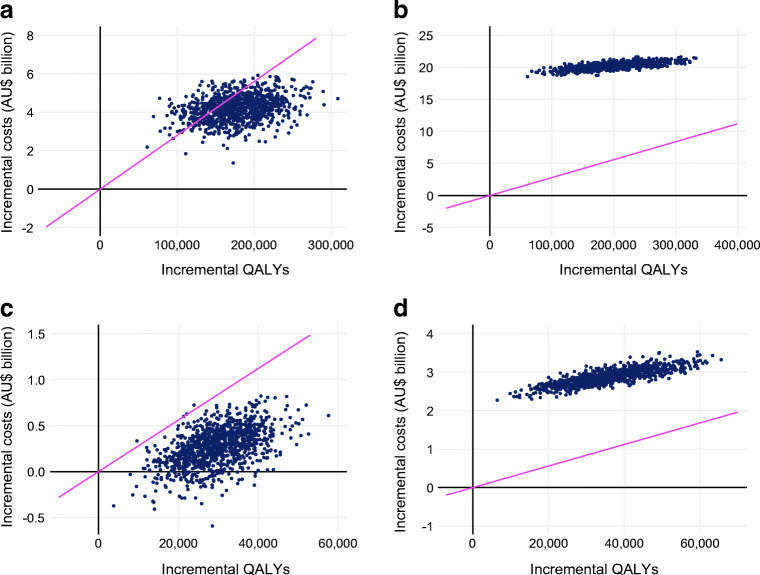
Results from probabilistic sensitivity analyses presented in a cost-effectiveness plane. Incremental costs and benefits are as compared with the current use for each scenario. The pink line represents the $AU28,000 per QALY willingness-to-pay threshold. All costs are in 2020 Australian dollars. All health economic outcomes have been subject to 5% annual discounting. (**a**) SGLT2i/total population, (**b**) GLP-1 RA/total population, (**c**) SGLT2i/secondary prevention population, (**d**) GLP-1 RA/secondary prevention population

The results of the probabilistic sensitivity analysis are shown in Fig. 4. For SGLT2i use, 75.4% and 99.6% of simulations produced cost-effective simulations for the total and secondary prevention populations, respectively. Furthermore, 9.8% of the simulations produced simulations where increased SGLT2i use was dominant over current use in the secondary prevention population. For GLP-1 RAs, none of the simulations produced a cost-effective ICER in either population.

## Discussion

### Principal findings

We modelled the effects and costs of widespread use of SGLT2is and GLP-1 RAs in the total and secondary prevention populations with type 2 diabetes in Australia, considering only their benefits on major cardiovascular and kidney outcomes, from 2020 to 2040. We found that, from a healthcare perspective, use of SGLT2is is probably cost-effective in both populations. Conversely, our analysis suggests that use of GLP-1 RAs is unlikely to be cost-effective from a healthcare or societal perspective (based solely on their cardiovascular benefits) in either population at current prices. While the findings for GLP-1 RAs have a more complex interpretation, the fact that use of SGLT2is is probably cost-effective in our analyses has important policy implications. In particular, our results suggest that the current reimbursement criteria that limit the use of SGLT2is among people with type 2 diabetes may need to be reconsidered.

### Cost-effectiveness of SGLT2is

Under the assumptions of our model, use of SGLT2is was cost-effective: SGLT2i use met the AU$28,000 per QALY willingness-to-pay threshold in most one-way sensitivity and scenario analyses in both populations, and also did so in most probabilistic sensitivity analyses, especially in the secondary prevention population. These findings suggest that the cardiovascular and kidney benefits of treatment with SGLT2is are worth the cost for all people with type 2 diabetes, regardless of use of other medications or their HbA_1c_ levels.

To our knowledge, this is the first cost-effectiveness analysis of its kind. Prior analyses have either used models that exclude the results of cardiovascular outcomes trials (with any reductions in cardiovascular and kidney outcomes being extrapolated only from changes in HbA_1c_ [13]), considered only specific high-risk populations [45, 46], or modelled the populations used in cardiovascular outcomes trials, which represent only 20–60% of all people with type 2 diabetes [14]. Most of these studies have found use of SGLT2is to be cost-effective. We extend these findings by showing that use of SGLT2is is cost-effective regardless of their effects on glucose levels, suggesting that existing restrictions on their use may not be justified from a health economic perspective. However, our results do not necessarily suggest that everyone with type 2 diabetes should receive an SGLT2i, nor do they address other considerations about widespread SGLT2i use.

### Cost-effectiveness of GLP-1 RAs

Interpretation of our findings for GLP-1 RAs is complex. The base-case analyses demonstrate that, when focusing solely on their cardiovascular benefits, GLP-1 RAs were not cost-effective in either the total or secondary prevention populations. Importantly, GLP-1 RAs cause weight loss, reduce hypoglycaemia (compared with sulfonylureas and insulin), and are one of the most effective glucose-lowering medications, which are effects that we have not incorporated [7]. Thus, our findings apply only to their cost-effectiveness in the whole type 2 diabetes population independently of these effects; analyses taking these effects into account on specific patient groups have shown GLP-1 RAs to be cost-effective [13]. Further cost-effectiveness analyses including both effects on glucose levels and CVD in broader populations are warranted. This is important because there is a high burden of CVD among people with diabetes, and, with ageing of the population, stroke in particular is becoming more frequent among people with type 2 diabetes [20]; these are outcomes that increased uptake of GLP-1 RAs could affect.

### Strengths and limitations

The primary strength of this analysis is the large, representative population on which the model is based. Unlike trial populations, this real-world population allowed us to estimate the cost-effectiveness of SGLT2is and GLP-1 RAs among the entire population with type 2 diabetes. Nevertheless, our findings should be interpreted in the context of several limitations. First, we assumed a uniform effect of the medications across all people with diabetes, even those who would not have been eligible for the cardiovascular outcome trials from which these effect estimates were derived. Importantly, there is evidence that the effects of both medications varies by disease stage [47, 48], although results from real-world studies of SGLT2is support their effectiveness on cardiovascular and kidney outcomes in broader populations [49, 50]. Importantly, clinical data are not readily available in Australia via linkage, and thus we could not assess eligibility for trials from which the HRs used in this study were derived.

Second, we have not considered several practical limitations and costs associated with increasing medication use, such as marketing and advocacy required to reach high rates of use. Third, we did not account for earlier disease stages, such as advanced chronic kidney disease, nor did we account for side-effects of these medications. Fourth, the chronic costs of diabetes that we used also included costs of hospitalisations; thus, we will have double-counted some hospitalisation costs. Nevertheless, even among people with diabetes and CVD, cardiovascular hospitalisations represent only a relatively small minority of all hospital admissions [51, 52]. Fifth, we did not estimate lifetime benefits and costs of these medications, which has implications for the interpretation of the age-stratified results; thus, our results should not be used to determine which age groups with diabetes should be deemed eligible for these medications. Sixth, it is probable that real-world adherence to these medications would be lower than in the trials used to inform our study [9], although it should be noted that the scenario analyses with lower uptake did not substantially affect the ICER. Finally, allocative efficiency decisions should not be based solely on cost-effectiveness analyses, as relative resource scarcity between healthcare domains is not accounted for. For example, the reduction in CVD associated with increased use of GLP-1 RAs may come at a relatively high cost, but this cost represents a shift from the human domain (i.e. the time and cost of nurses, doctors and administrators) to medication cost, which may be desirable.

### Conclusions

Use of SGLT2is is probably cost-effective among all people with type 2 diabetes, especially among those with pre-existing CVD. Conversely, at current prices, GLP-1 RAs are unlikely to meet the arbitrary AU$28,000 per QALY willingness-to-pay threshold in either the total or secondary prevention population when considering only their benefits on CVD.

## Supplementary Information

ESM (PDF 1782 kb)

## Data Availability

Because the model population is derived from de-identified registry data, the data cannot be made available owing to privacy concerns.

## Abbreviations

ESKD: End-stage kidney disease
GLP-1 RA: Glucagon-like peptide-1 receptor agonist
HF: Heart failure
ICER: Incremental cost-effectiveness ratio
MI: Myocardial infarction
NDSS: National Diabetes Services Scheme
QALY: Quality-adjusted life year
SGLT2i: Sodium–glucose co-transporter 2 inhibitor
