# Supplementary material for: Projecting the incidence and costs of major cardiovascular and kidney complications of type 2 diabetes with widespread SGLT2i and GLP-1 RA use: a cost-effectiveness analysis
Source: Diabetologia. 2022 Nov 21;66(4):642–56. doi: 10.1007/s00125-022-05832-0 (PMC9947091; doi:10.1007/s00125-022-05832-0)
Supplement: Supplementary file 1 — (PDF 1782 kb) [file 125_2022_5832_MOESM1_ESM.pdf]

Projecting the incidence and costs of major cardiovascular and kidney complications of type 2 diabetes with widespread SGLT2i and GLP-1 RA use: A cost-effectiveness analysis

Electronic Supplementary Material

ESM Methods

Diabetes incidence in the model.

Age-specific diabetes incidence rates were modelled using the National Diabetes Services Scheme population. All incident cases of type 2 diabetes from 2017-2019 were included as the numerator. The denominator was all people without diabetes in Victoria, New South Wales, Queensland, and the Australian Capital Territory, derived from (1). This data was tabulated into incident cases per population by single year of age and calendar year. A Poisson model was then used to analyse the incidence of diabetes, with spline effects of age and a linear effect of calendar time, using log-population size as the offset. Models were fit for men and women separately. These models were then used to predict the incidence of type 2 diabetes at each single year of age, with the prediction year set at 2019. The age-specific incidence of type 2 diabetes by sex is shown in ESM Fig. 1. The proportion of people with incident type 2 diabetes and prior myocardial infarction, stroke, heart failure, and end-stage kidney disease was estimated using a lookback period from 30 June 2019 to 1 July 2010. Both diabetes incidence and the proportion of incident cases with prior myocardial infarction, stroke, heart failure, and end-stage kidney disease was assumed to remain constant from 2020-2040. The number of incident cases of type 2 diabetes from 2020-2040 was estimated using these incidence rates and the Australian Bureau of Statistics Australian Population Projections, medium series (2).

References

1. The Australian Bureau of Statistics. Naitonal, state, and territory population. Available at: https://www.abs.gov.au/statistics/people/population/national-state-and-territory-population/jun-2020 [Accessed 3.11.2021].
2. The Australian Bureau of Statistics. Population Projections, Australia. Available at: https://www.abs.gov.au/statistics/people/population/population-projections-australia/2017-base-2066 [Accessed 3.11.2021].

Transition probabilities for the model.

We used a linked dataset derived from the National Diabetes Services Scheme (NDSS) to estimate the incidence of hospitalization for myocardial infarction (MI), stroke, and heart failure (HF), the incidence of end-stage kidney disease (ESKD), and all-cause mortality. MI, stroke, and HF admission was defined from hospital admissions data when one of the following International Classification of Diseases (ICD)-10 codes was listed as the primary diagnosis for an admission: MI (ICD-10 codes: I21-I22), stroke (I60-I64), and HF (I110, I130, I132, or I50). We included admissions to public hospitals only. ESKD was defined from the Australia and New Zealand Dialysis and Transplant Registry as initiation of kidney replacement therapy (receipt of dialysis or a kidney transplant). All-cause mortality was derived from the National Death Index.

Incidence rates were estimated for the period covering 1 July 2016 to 31 May 2019. We included a look-back period from 1 July 2010 to 30 June 2016 to split the NDSS population into their appropriate health state (i.e., whether they had had a prior admission for MI, stroke, or HF, or developed ESKD). This was used to split subsequent follow-up time by the presence or absence of prior MI, stroke, HF, and/or ESKD. If an individual experienced any of these outcomes during follow-up, their risk time was split into periods representing time before and after this event. This way, we could estimate the effect of each prior event on subsequent events. To partition follow-up time, individuals were followed from 1 July 2016, date of NDSS registration, or migration into one of the four states in this study, until an event (MI, stroke, HF, ESKD, or death), migration out of one of the four states, or end of follow-up. Except for death, once an event occurred, individuals were followed subsequently until another event, migration out, or end of follow-up. Because admissions are only recorded in hospital admitted datasets at discharge, we terminated follow-up on 31 May 2019 to avoid underestimating admission rates.

Follow-up time was then split into 6-month intervals by attained age (10-100 years), duration of diabetes (0-50 years), and calendar time, and event counts and risk time were then tabulated in these intervals, with each tabulation assigned the midpoint of the interval. Age at diagnosis of diabetes was added to the tabulation as attained age minus duration of diabetes. Incidence and mortality rates were then analyzed via a Poisson model (one for each outcome) with spline effects of attained age, duration of diabetes, age at diagnosis of diabetes, a linear effect of calendar time, and binary effects of prior MI, stroke, HF, and ESKD, using log-person-time as the offset. Models were fit for men and women separately. The models were then used to predict the incidence of each complication by any combination of age, duration of diabetes, and prior MI, stroke, HF, and ESKD, with the prediction year set at 2019; however, the effect of calendar time was partially carried forward from 2020-2040. While the use of SGLT2is and GLP-1 RAs was relatively low during the estimation period, we nevertheless corrected these incidence rates for use of SGLT2is and GLP-1 RAs during the estimation period.

Examples of these incidence rates are shown in ESM Fig. 2-3. While the examples only show selected rates, we included predicted incidence rates for all possible sex, age, and duration of diabetes combinations in all health states for the model.

Validation of transition probabilities and model structure

We validated the transition probabilities and model structure using two tests. First, we compared the actual vs. predicted number of events using the actual population structure among people with type 2 diabetes. Events were predicted using the transition probabilities described above and actual demographic structure of the population with type 2 diabetes for three financial years (we selected 3 so that the first 6 years could be used to define prior events). This test (ESM Fig. 4-8) demonstrated that the modelled transition probabilities could recapitulate reality reasonably well in the absence of any other model inputs.

Second, we compared the actual vs. predicted number of events using the actual population structure in the first year, then a modelled population structure in the second 2 years. I.e., we tested our full model over the years financial years 2016-17 to 2018-19 to see if the model could recapitulate reality for years in which we had data. This test (ESM Fig. 9) demonstrated that our full model could recapitulate reality reasonably well.

ESM Table 1 – Characteristics of the National Diabetes Services Scheme population.

Number		951,300
Male		519,094 (54.6%)
Age at diagnosis of diabetes		57.0 (47.8, 65.8)
Duration of diabetes		10.2 (5.3, 16.6)
Age		68.9 (59.0, 77.6)
Age group	0-19	371 (0.0%)
	20-39	26,482 (2.8%)
	40-59	238,773 (25.1%)
	60-79	545,658 (57.4%)
	80+	206,727 (21.7%)
Prior MI		29,831
Prior stroke		17,299
Prior HHF		21,106
Prior ESKD		5929
MI during follow-up		21,006
Stroke during follow-up		15,036
HHF during follow-up		35,177
ESKD during follow-up		2609

Prior refers to the period between 1 July 2010 and 30 June 2016. Follow-up is the period from 1 July 2016 to 30 June 2019.

Abbreviations: MI – myocardial infarction; HHF – hospitalisation for heart failure; ESKD – end-stage kidney disease.

ESM Table 2 – Results from the base-case analysis, males only.

Total population					
	Current use	SGLT2i use		GLP-1 RA use	
		Absolute value	Difference to current use	Absolute value	Difference to current use
Myocardial infarctions	196,630	187,774	-8856 (-4.5%)	185,665	-10,965 (-5.6%)
Strokes	118,623	119,567	944 (0.8%)	105,664	-12,959 (-10.9%)
Hospitalisations for heart failure	278,492	212,364	-66,128 (-23.7%)	257,596	-20,896 (-7.5%)
End-stage kidney disease	23,771	19,147	-4625 (-19.5%)	23,941	170 (0.7%)
Deaths	611,293	590,012	-21,281 (-3.5%)	587,867	-23,426 (-3.8%)
Years of life lived	1,4911,252	15,144,833	233,580 (1.6%)	15,177,918	266,665 (1.8%)
Quality-adjusted life years	7,206,180	7,308,752	102,572 (1.4%)	7,322,676	116,495 (1.6%)
Acute healthcare costs	$7,643,904,768	$7,037,588,208	-$606,316,560	$7,163,171,616	-$480,733,152
Chronic healthcare costs	$49,898,797,184	$48,835,062,912	-$1,063,734,272	$50,327,847,040	$429,049,856
SGLT2i costs	$1,335,076,076	$5,081,731,040	$3,746,654,964	$1,356,149,312	$21,073,236
GLP-1 RA costs	$787,860,842	$799,694,136	$11,833,294	$12,004,450,080	$11,216,589,238
Total healthcare costs	$59,665,638,870	$61,754,076,296	2,088,437,426 (3.5%)	$70,851,618,048	11,185,979,178 (18.7%)
Acute absenteeism costs	$462,392,554	$441,859,997	-$20,532,557	$420,868,149	-$41,524,405
Chronic absenteeism costs	$2,850,596,336	$2,852,373,376	$1,777,040	$2,854,723,280	$4,126,944
Non-participation costs - morbidity	$34,617,438,464	$34,575,720,576	-$41,717,888	$34,638,219,520	$20,781,056
Non-participation costs - mortality	$10,608,157,176	$9,871,775,784	-$736,381,392	$9,758,685,208	-$849,471,968
Total productivity costs	$48,538,584,530	$47,741,729,733	-$796,854,797	$47,672,496,157	-$866,088,373
Total societal costs	$108,204,223,400	$109,495,806,029	$1,291,582,629	$118,524,114,205	$10,319,890,805
ICER– years of life lived	.	.	8941	.	41,948
ICER – QALYs	.	.	20,361	.	96,021
SICER – years of life lived	.	.	5530	.	38,700
SICER – QALYs	.	.	12,592	.	88,586

Secondary prevention population					
	Current use	SGLT2i use		GLP-1 RA use	
		Absolute value	Difference to current use	Absolute value	Difference to current use
Myocardial infarctions	99,803	96,152	-3651 (-3.7%)	95,547	-4256 (-4.3%)
Strokes	53,576	54,255	679 (1.3%)	48,566	-5010 (-9.4%)
Hospitalisations for heart failure	197,226	158,309	-38,918 (-19.7%)	186,530	-10,697 (-5.4%)
End-stage kidney disease	9105	7518	-1587 (-17.4%)	9336	231 (2.5%)
Deaths	210,514	205,987	-4527 (-2.2%)	205,296	-5218 (-2.5%)
Years of life lived	2,349,664	2,399,861	50,197 (2.1%)	2,410,608	60,944 (2.6%)
Quality-adjusted life years	955,000	973,826	18,826 (2.0%)	977,735	22,734 (2.4%)
Acute healthcare costs	$3,547,367,392	$3,253,504,904	-$293,862,488	$3,368,592,112	-$178,775,280
Chronic healthcare costs	$15,364,855,040	$15,248,132,800	-$116,722,240	$15,671,453,184	$306,598,144
SGLT2i costs	$199,053,025	$762,665,630	$563,612,605	$203,793,071	$4,740,046
GLP-1 RA costs	$117,466,029	$120,018,009	$2,551,980	$1,803,948,680	$1,686,482,652
Total healthcare costs	$19,228,741,486	$19,384,321,343	155,579,857 (0.8%)	$21,047,787,047	1,819,045,561 (9.5%)
Acute absenteeism costs	$144,293,341	$137,761,989	-$6,531,352	$133,286,137	-$11,007,204
Chronic absenteeism costs	$413,115,339	$415,330,826	$2,215,487	$415,484,060	$2,368,721
Non-participation costs - morbidity	$6,185,578,112	$6,207,136,496	$21,558,384	$6,219,772,096	$34,193,984
Non-participation costs - mortality	$2,020,059,186	$1,897,828,206	-$122,230,980	$1,882,322,811	-$137,736,375
Total productivity costs	$8,763,045,978	$8,658,057,517	-$104,988,461	$8,650,865,104	-$112,180,874
Total societal costs	$27,991,787,463	$28,042,378,859	$50,591,396	$29,698,652,151	$1,706,864,688
ICER– years of life lived	.	.	3099	.	29,848
ICER – QALYs	.	.	8264	.	80,013
SICER – years of life lived	.	.	1008	.	28,007
SICER – QALYs	.	.	2687	.	75,078

All costs are presented in 2020 Australian dollars. All health economic outcomes have been subject to 5% annual discounting.

Abbreviations: SGLT2i – sodium-glucose co-transporter 2 inhibitor; GLP-1 RA – glucagon-like peptide 1 receptor agonist; ICER – incremental cost-effectiveness ratio; QALY – Quality Adjusted Life Year.

ESM Table 3 – Results from the base-case analysis, females only.

Total population					
	Current use	SGLT2i use		GLP-1 RA use	
		Absolute value	Difference to current use	Absolute value	Difference to current use
Myocardial infarctions	97,794	93,273	-4520 (-4.6%)	92,304	-5490 (-5.6%)
Strokes	68,852	69,271	419 (0.6%)	61,402	-7450 (-10.8%)
Hospitalisations for heart failure	214,204	163,092	-51,113 (-23.9%)	198,000	-16,204 (-7.6%)
End-stage kidney disease	11,047	8801	-2246 (-20.3%)	11,048	1 (0.0%)
Deaths	444,426	429,718	-14,708 (-3.3%)	427,935	-16,491 (-3.7%)
Years of life lived	11,947,350	12,113,788	166,438 (1.4%)	12,140,713	193,362 (1.6%)
Quality-adjusted life years	5,835,233	5,909,107	73,874 (1.3%)	5,919,669	84,436 (1.4%)
Acute healthcare costs	$5,118,277,008	$4,678,827,264	-$439,449,744	$4,803,511,552	-$314,765,456
Chronic healthcare costs	$34,711,532,800	$34,212,428,672	-$499,104,128	$34,988,147,072	$276,614,272
SGLT2i costs	$1,081,595,956	$4,108,260,240	$3,026,664,284	$1,096,943,020	$15,347,064
GLP-1 RA costs	$638,276,072	$646,502,470	$8,226,398	$9,709,990,976	$9,071,714,904
Total healthcare costs	$41,549,681,836	$43,646,018,646	2,096,336,810 (5.0%)	$50,598,592,620	9,048,910,784 (21.8%)
Acute absenteeism costs	$115,061,524	$110,500,494	-$4,561,030	$104,615,853	-$10,445,671
Chronic absenteeism costs	$1,368,027,632	$1,370,519,868	$2,492,236	$1,371,393,408	$3,365,776
Non-participation costs - morbidity	$4,605,820,624	$4,450,168,304	-$155,652,320	$4,478,809,808	-$127,010,816
Non-participation costs - mortality	$3,112,705,054	$2,896,534,246	-$216,170,808	$2,858,574,480	-$254,130,574
Total productivity costs	$9,201,614,834	$8,827,722,912	-$373,891,922	$8,813,393,549	-$388,221,285
Total societal costs	$50,751,296,670	$52,473,741,558	$1,722,444,888	$59,411,986,169	$8,660,689,499
ICER– years of life lived	.	.	12,595	.	46,798
ICER – QALYs	.	.	28377	.	107,169
SICER – years of life lived	.	.	10,349	.	44,790
SICER – QALYs	.	.	23,316	.	102,571

Secondary prevention population					
	Current use	SGLT2i use		GLP-1 RA use	
		Absolute value	Difference to current use	Absolute value	Difference to current use
Myocardial infarctions	46,914	45,163	-1751 (-3.7%)	44,959	-1955 (-4.2%)
Strokes	26,498	26,815	317 (1.2%)	24,041	-2457 (-9.3%)
Hospitalisations for heart failure	143,772	116,200	-27,573 (-19.2%)	136,233	-7540 (-5.2%)
End-stage kidney disease	4110	3373	-737 (-17.9%)	4192	82 (2.0%)
Deaths	135,247	132,774	-2473 (-1.8%)	132,208	-3039 (-2.2%)
Years of life lived	1,367,791	1,395,850	28,059 (2.1%)	1,403,874	36,083 (2.6%)
Quality-adjusted life years	547,744	558,275	10,531 (1.9%)	561,099	13,355 (2.4%)
Acute healthcare costs	$2,178,266,312	$1,981,260,192	-$197,006,120	$2,074,286,616	-$103,979,696
Chronic healthcare costs	$8,332,686,080	$8,304,237,600	-$28,448,480	$8,505,713,696	$173,027,616
SGLT2i costs	$116,417,871	$445,527,254	$329,109,384	$119,255,416	$2,837,546
GLP-1 RA costs	$68,701,016	$70,111,057	$1,410,041	$1,055,632,804	$986,931,788
Total healthcare costs	$10,696,071,278	$10,801,136,103	105,064,824 (1.0%)	$11,754,888,532	1,058,817,254 (9.9%)
Acute absenteeism costs	$16,765,475	$15,974,083	-$791,391	$15,509,980	-$1,255,495
Chronic absenteeism costs	$76,038,880	$76,338,993	$300,113	$76,361,855	$322,975
Non-participation costs - morbidity	$2,941,035,696	$2,951,555,720	$10,520,024	$2,954,019,840	$12,984,144
Non-participation costs - mortality	$388,699,466	$364,475,931	-$24,223,535	$361,615,040	-$27,084,427
Total productivity costs	$3,422,539,517	$3,408,344,727	-$14,194,790	$3,407,506,715	-$15,032,802
Total societal costs	$14,118,610,795	$14,209,480,829	$90,870,035	$15,162,395,247	$1,043,784,452
ICER– years of life lived	.	.	3744	.	29,344
ICER – QALYs	.	.	9977	.	79,280
SICER – years of life lived	.	.	3239	.	28,927
SICER – QALYs	.	.	8629	.	78,154

All costs are presented in 2020 Australian dollars. All health economic outcomes have been subject to 5% annual discounting.

Abbreviations: SGLT2i – sodium-glucose co-transporter 2 inhibitor; GLP-1 RA – glucagon-like peptide 1 receptor agonist; ICER – incremental cost-effectiveness ratio; QALY – Quality Adjusted Life Year.

ESM Table 4 – Results from the base-case analysis ages 10-39 only. Note – these are not lifetime projections, only events that happen while an individual is within the age group.

Total population					
	Current use	SGLT2i use		GLP-1 RA use	
		Absolute value	Difference to current use	Absolute value	Difference to current use
Myocardial infarctions	903	856	-47 (-5.3%)	839	-64 (-7.1%)
Strokes	442	437	-5 (-1.2%)	389	-53 (-12.0%)
Hospitalisations for heart failure	1188	937	-251 (-21.1%)	1087	-101 (-8.5%)
End-stage kidney disease	468	377	-91 (-19.4%)	466	-1 (-0.3%)
Deaths	1410	1312	-98 (-6.9%)	1292	-118 (-8.3%)
Years of life lived	672,040	672,380	340 (0.1%)	672,452	412 (0.1%)
Quality-adjusted life years	357,809	358,060	251 (0.1%)	358,055	246 (0.1%)
Acute healthcare costs	$27,041,108	$24,437,225	-$2,603,883	$24,732,147	-$2,308,961
Chronic healthcare costs	$1,636,255,456	$1,614,127,444	-$22,128,012	$1,635,185,516	-$1,069,940
SGLT2i costs	$61,098,507	$229,321,999	$168,223,492	$61,133,388	$34,881
GLP-1 RA costs	$36,055,715	$36,087,597	$31,882	$541,144,479	$505,088,765
Total healthcare costs	$1,760,450,786	$1,903,974,265	143,523,479 (8.2%)	$2,262,195,530	501,744,744 (28.5%)
Acute absenteeism costs	$10,061,453	$9,603,044	-$458,410	$9,151,701	-$909,753
Chronic absenteeism costs	$292,558,339	$292,439,564	-$118,775	$292,555,997	-$2,342
Non-participation costs - morbidity	$1,134,478,256	$1,129,269,784	-$5,208,472	$1,133,751,544	-$726,712
Non-participation costs - mortality	$169,084,516	$157,462,179	-$11,622,337	$154,975,295	-$14,109,221
Total productivity costs	$1,606,182,564	$1,588,774,571	-$17,407,994	$1,590,434,537	-$15,748,028
Total societal costs	$3,366,633,350	$3,492,748,835	$126,115,485	$3,852,630,067	$485,996,717
ICER– years of life lived	.	.	422,210	.	1,217,486
ICER – QALYs	.	.	571,551	.	2,039,289
SICER – years of life lived	.	.	371,000	.	1,179,273

SICER – QALYs	.	.	502,227	.	1,975,283
**Secondary prevention population**					
	Current use	SGLT2i use		GLP-1 RA use	
		Absolute value	Difference to current use	Absolute value	Difference to current use
Myocardial infarctions	76	72	-4 (-5.2%)	71	-5 (-6.7%)
Strokes	24	24	-0 (-1.2%)	21	-3 (-11.5%)
Hospitalisations for heart failure	242	196	-46 (-19.0%)	224	-18 (-7.5%)
End-stage kidney disease	45	36	-8 (-18.8%)	45	0 (0.3%)
Deaths	66	61	-4 (-6.5%)	61	-5 (-7.5%)
Years of life lived	12,267	12,279	12 (0.1%)	12,281	14 (0.1%)
Quality-adjusted life years	5485	5492	7 (0.1%)	5491	6 (0.1%)
Acute healthcare costs	$2,686,393	$2,343,787	-$342,606	$2,482,081	-$204,312
Chronic healthcare costs	$79,129,650	$77,747,854	-$1,381,796	$79,214,875	$85,225
SGLT2i costs	$1,057,372	$3,975,719	$2,918,348	$1,058,486	$1114
GLP-1 RA costs	$623,981	$625,645	$1664	$9,369,571	$8,745,590
Total healthcare costs	$83,497,395	$84,693,005	1,195,610 (1.4%)	$92,125,013	8,627,618 (10.3%)
Acute absenteeism costs	$782,998	$735,076	-$47,922	$719,224	-$63,774
Chronic absenteeism costs	$9,527,661	$9,541,912	$14,251	$9,538,144	$10,483
Non-participation costs - morbidity	$47,062,991	$46,820,232	-$242,759	$47,107,657	$44,666
Non-participation costs - mortality	$6,663,269	$6,240,525	-$422,744	$6,161,801	-$501,468
Total productivity costs	$64,036,919	$63,337,745	-$699,174	$63,526,825	-$510,094
Total societal costs	$147,534,314	$148,030,750	$496,436	$155,651,839	$8,117,524
ICER– years of life lived	.	.	100,902	.	618,776
ICER – QALYs	.	.	179,399	.	1,344,138
SICER – years of life lived	.	.	41,896	.	582,192
SICER – QALYs	.	.	74,489	.	1264,668

All costs are presented in 2020 Australian dollars. All health economic outcomes have been subject to 5% annual discounting.

Abbreviations: SGLT2i – sodium-glucose co-transporter 2 inhibitor; GLP-1 RA – glucagon-like peptide 1 receptor agonist; ICER – incremental cost-effectiveness ratio; QALY – Quality Adjusted Life Year.

ESM Table 5 – Results from the base-case analysis ages 40-59 only. Note – these are not lifetime projections, only events that happen while an individual is within the age group.

Total population					
	Current use	SGLT2i use		GLP-1 RA use	
		Absolute value	Difference to current use	Absolute value	Difference to current use
Myocardial infarctions	34,908	32,878	-2029 (-5.8%)	32,358	-2549 (-7.3%)
Strokes	14,262	14,075	-187 (-1.3%)	12,522	-1740 (-12.2%)
Hospitalisations for heart failure	23,965	18,566	-5399 (-22.5%)	21,781	-2184 (-9.1%)
End-stage kidney disease	7110	5706	-1404 (-19.7%)	7078	-32 (-0.4%)
Deaths	35,497	32,971	-2527 (-7.1%)	32,611	-2886 (-8.1%)
Years of life lived	5,826,833	5,838,283	11,450 (0.2%)	5,840,189	13,356 (0.2%)
Quality-adjusted life years	2,982,569	2,989,414	6846 (0.2%)	2,989,982	7413 (0.2%)
Acute healthcare costs	$771,047,612	$704,508,402	-$66,539,210	$704,465,182	-$66,582,430
Chronic healthcare costs	$17,103,519,104	$16,682,545,792	-$420,973,312	$17,076,299,392	-$27,219,712
SGLT2i costs	$530,741,893	$1,995,648,168	$1,464,906,275	$531,848,750	$1,106,857
GLP-1 RA costs	$313,203,696	$314,048,133	$844,437	$4,707,853,184	$4,394,649,488
Total healthcare costs	$18,718,512,305	$19,696,750,495	978,238,190 (5.2%)	$23,020,466,508	4,301,954,203 (23.0%)
Acute absenteeism costs	$306,415,079	$292,660,707	-$13,754,372	$278,558,722	-$27,856,357
Chronic absenteeism costs	$2,455,667,440	$2,455,278,576	-$388,864	$2,456,421,088	$753,648
Non-participation costs - morbidity	$17,346,443,712	$17,279,776,512	-$66,667,200	$17,339,140,480	-$7,303,232
Non-participation costs - mortality	$5,309,058,524	$4,939,610,432	-$369,448,092	$4,875,576,936	-$433,481,588
Total productivity costs	$25,417,584,755	$24,967,326,227	-$450,258,528	$24,949,697,226	-$467,887,529
Total societal costs	$44,136,097,060	$44,664,076,722	$527,979,662	$47,970,163,734	$3,834,066,674
ICER– years of life lived	.	.	85,433	.	322,088
ICER – QALYs	.	.	142,900	.	580,326
SICER – years of life lived	.	.	46,110	.	287,057

SICER – QALYs	.	.	77,127	.	517,209
**Secondary prevention population**					
	Current use	SGLT2i use		GLP-1 RA use	
		Absolute value	Difference to current use	Absolute value	Difference to current use
Myocardial infarctions	11,137	10,556	-581 (-5.2%)	10,440	-697 (-6.3%)
Strokes	3129	3091	-37 (-1.2%)	2776	-353 (-11.3%)
Hospitalisations for heart failure	10,220	8241	-1979 (-19.4%)	9449	-771 (-7.5%)
End-stage kidney disease	1779	1450	-329 (-18.5%)	1788	10 (0.5%)
Deaths	5834	5463	-372 (-6.4%)	5428	-406 (-7.0%)
Years of life lived	418,759	420,148	1389 (0.3%)	420,313	1553 (0.4%)
Quality-adjusted life years	183,098	183,747	649 (0.4%)	183,783	685 (0.4%)
Acute healthcare costs	$212,211,875	$192,961,976	-$19,249,899	$196,503,938	-$15,707,938
Chronic healthcare costs	$2,923,504,808	$2,861,698,376	-$61,806,432	$2,932,403,488	$8,898,680
SGLT2i costs	$36,105,075	$136,140,751	$100,035,676	$36,228,699	$123,625
GLP-1 RA costs	$21,306,483	$21,423,991	$117,508	$320,691,542	$299,385,059
Total healthcare costs	$3,193,128,240	$3,212,225,093	19,096,853 (0.6%)	$3,485,827,667	292,699,426 (9.2%)
Acute absenteeism costs	$80,685,326	$76,941,614	-$3,743,712	$74,380,785	-$6,304,541
Chronic absenteeism costs	$279,466,989	$280,449,292	$982,303	$280,443,535	$976,546
Non-participation costs - morbidity	$2,501,852,856	$2,501,213,608	-$639,248	$2,509,434,312	$7,581,456
Non-participation costs - mortality	$707,620,121	$663,295,157	-$44,324,964	$657,416,716	-$50,203,406
Total productivity costs	$3,569,625,292	$3,521,899,671	-$47,725,621	$3,521,675,347	-$47,949,945
Total societal costs	$6,762,753,532	$6,734,124,765	-$28,628,767	$7,007,503,014	$244,749,482
ICER– years of life lived	.	.	13,751	.	188,444
ICER – QALYs	.	.	29,420	.	427,259
SICER – years of life lived	.	.	Dominant	.	157,573
SICER – QALYs	.	.	Dominant	.	357,266

All costs are presented in 2020 Australian dollars. All health economic outcomes have been subject to 5% annual discounting.

Abbreviations: SGLT2i – sodium-glucose co-transporter 2 inhibitor; GLP-1 RA – glucagon-like peptide 1 receptor agonist; ICER – incremental cost-effectiveness ratio; QALY – Quality Adjusted Life Year.

ESM Table 6 – Results from the base-case analysis ages 60-79 only. Note – these are not lifetime projections, only events that happen while an individual is within the age group.

Total population					
	Current use	SGLT2i use		GLP-1 RA use	
		Absolute value	Difference to current use	Absolute value	Difference to current use
Myocardial infarctions	150,044	141,491	-8553 (-5.7%)	139,895	-10,149 (-6.8%)
Strokes	92,287	91,613	-674 (-0.7%)	81,181	-11,106 (-12.0%)
Hospitalisations for heart failure	232,422	176,165	-56,257 (-24.2%)	211,377	-21,045 (-9.1%)
End-stage kidney disease	23,747	19,018	-4729 (-19.9%)	23,827	79 (0.3%)
Deaths	352,658	329,521	-23,137 (-6.6%)	327,225	-25,433 (-7.2%)
Years of life lived	14,382,011	14,511,244	129,232 (0.9%)	14,527,517	145,506 (1.0%)
Quality-adjusted life years	6,997,179	7,059,168	61,989 (0.9%)	7,065,606	68,427 (1.0%)
Acute healthcare costs	$5,438,278,944	$4,886,220,672	-$552,058,272	$4,984,636,608	-$453,642,336
Chronic healthcare costs	$48,131,948,544	$46,790,219,136	-$1,341,729,408	$48,258,876,928	$126,928,384
SGLT2i costs	$1,300,805,444	$4,923,304,320	$3,622,498,876	$1,312,538,288	$11,732,844
GLP-1 RA costs	$767,636,920	$774,763,072	$7,126,152	$11,618,411,296	$10,850,774,376
Total healthcare costs	$55,638,669,852	$57,374,507,200	1,735,837,348 (3.1%)	$66,174,463,120	10,535,793,268 (18.9%)
Acute absenteeism costs	$260,977,541	$250,096,743	-$10,880,799	$237,773,575	-$23,203,967
Chronic absenteeism costs	$1,470,398,180	$1,475,175,116	$4,776,936	$1,477,139,608	$6,741,428
Non-participation costs - morbidity	$20,742,337,280	$20,616,842,880	-$125,494,400	$20,644,137,024	-$98,200,256
Non-participation costs - mortality	$8,242,719,208	$7,671,237,448	-$571,481,760	$7,586,707,376	-$656,011,832
Total productivity costs	$30,716,432,209	$30,013,352,187	-$703,080,023	$29,945,757,583	-$770,674,627
Total societal costs	$86,355,102,061	$87,387,859,387	$1,032,757,326	$96,120,220,703	$9,765,118,642
ICER– years of life lived	.	.	13,432	.	72,408
ICER – QALYs	.	.	28,002	.	153,971
SICER – years of life lived	.	.	7991	.	67,111

SICER – QALYs	.	.	16,660	.	142,708
**Secondary prevention population**					
	Current use	SGLT2i use		GLP-1 RA use	
		Absolute value	Difference to current use	Absolute value	Difference to current use
Myocardial infarctions	73,426	69,929	-3497 (-4.8%)	69,476	-3950 (-5.4%)
Strokes	36,474	36,381	-93 (-0.3%)	32,607	-3867 (-10.6%)
Hospitalisations for heart failure	152,721	122,577	-30,144 (-19.7%)	142,399	-10,322 (-6.8%)
End-stage kidney disease	9673	7961	-1711 (-17.7%)	9879	206 (2.1%)
Deaths	105,129	100,052	-5078 (-4.8%)	99,304	-5826 (-5.5%)
Years of life lived	2,039,191	2,065,907	26,716 (1.3%)	2,069,807	30,616 (1.5%)
Quality-adjusted life years	832122	842644	10,521 (1.3%)	843,987	11,864 (1.4%)
Acute healthcare costs	$2,383,714,216	$2,141,529,832	-$242,184,384	$2,222,781,896	-$160,932,320
Chronic healthcare costs	$13,964,117,440	$13,723,337,504	-$240,779,936	$14,134,341,056	$170,223,616
SGLT2i costs	$172,860,138	$657,776,766	$484,916,629	$175,243,448	$2,383,310
GLP-1 RA costs	$102,008,969	$103,512,016	$1,503,047	$1,551,231,244	$1,449,222,275
Total healthcare costs	$16,622,700,763	$16,626,156,118	3,455,355 (0.0%)	$18,083,597,644	1,460,896,881 (8.8%)
Acute absenteeism costs	$79,590,492	$76,059,382	-$3,531,110	$73,696,108	-$5,894,384
Chronic absenteeism costs	$200,159,565	$201,678,615	$1,519,050	$201,864,236	$1,704,672
Non-participation costs - morbidity	$6,577,697,920	$6,610,658,368	$32,960,448	$6,617,250,016	$39,552,096
Non-participation costs - mortality	$1,694,475,256	$1,592,768,446	-$101,706,810	$1,580,359,330	-$114,115,926
Total productivity costs	$8,551,923,232	$8,481,164,811	-$70,758,422	$8,473,169,690	-$78,753,542
Total societal costs	$25,174,623,995	$25,107,320,928	-$67,303,067	$26,556,767,334	$1,382,143,339
ICER– years of life lived	.	.	129	.	47,717
ICER – QALYs	.	.	328	.	123,135
SICER – years of life lived	.	.	Dominant	.	45,145
SICER – QALYs	.	.	Dominant	.	116,497

All costs are presented in 2020 Australian dollars. All health economic outcomes have been subject to 5% annual discounting.

Abbreviations: SGLT2i – sodium-glucose co-transporter 2 inhibitor; GLP-1 RA – glucagon-like peptide 1 receptor agonist; ICER – incremental cost-effectiveness ratio; QALY – Quality Adjusted Life Year.

ESM Table 7 – Results from the base-case analysis ages 80+ only. Note – these are not lifetime projections, only events that happen while an individual is within the age group.

**Total population**					
	Current use	SGLT2i use		GLP-1 RA use	
		Absolute value	Difference to current use	Absolute value	Difference to current use
Myocardial infarctions	108,569	105,822	-2747 (-2.5%)	104,876	-3693 (-3.4%)
Strokes	80,484	82,713	2229 (2.8%)	72,973	-7511 (-9.3%)
Hospitalisations for heart failure	235,121	179,787	-55,334 (-23.5%)	221,352	-13,770 (-5.9%)
End-stage kidney disease	3494	2847	-647 (-18.5%)	3618	124 (3.6%)
Deaths	666,154	655,926	-10,228 (-1.5%)	654,674	-11,480 (-1.7%)
Years of life lived	5,977,719	6,236,714	258,996 (4.3%)	6,278,472	300,753 (5.0%)
Quality-adjusted life years	2,703,857	2,811,217	107,360 (4.0%)	2,828,703	124,845 (4.6%)
Acute healthcare costs	$6,525,814,032	$6,101,249,120	-$424,564,912	$6,252,849,216	-$272,964,816
Chronic healthcare costs	$17,738,606,976	$17,960,598,912	$221,991,936	$18,345,631,104	$607,024,128
SGLT2i costs	$524,026,184	$2,041,716,856	$1,517,690,672	$547,571,892	$23,545,708
GLP-1 RA costs	$309,240,586	$321,297,801	$12,057,215	$4,847,032,336	$4,537,791,750
Total healthcare costs	$25,097,687,778	$26,424,862,689	1,327,174,911 (5.3%)	$29,993,084,548	4,895,396,770 (19.5%)
Acute absenteeism costs	$0	$0	$0	$0	$0
Chronic absenteeism costs	$0	$0	$0	$0	$0
Non-participation costs - morbidity	$0	$0	$0	$0	$0
Non-participation costs - mortality	$0	$0	$0	$0	$0
Total productivity costs	$0	$0	$0	$0	$0
Total societal costs	$25,097,687,778	$26,424,862,689	$1,327,174,911	$29,993,084,548	$4,895,396,770
ICER– years of life lived	.	.	5124	.	16,277
ICER – QALYs	.	.	12,362	.	39,212
SICER – years of life lived	.	.	5124	.	16,277

SICER – QALYs	.	.	12,362	.	39,212
**Secondary prevention population**					
	Current use	SGLT2i use		GLP-1 RA use	
		Absolute value	Difference to current use	Absolute value	Difference to current use
Myocardial infarctions	62,078	60,757	-1321 (-2.1%)	60,519	-1559 (-2.5%)
Strokes	40,447	41,573	1126 (2.8%)	37,203	-3244 (-8.0%)
Hospitalisations for heart failure	177,816	143,494	-34,322 (-19.3%)	170,691	-7125 (-4.0%)
End-stage kidney disease	1719	1443	-276 (-16.0%)	1816	97 (5.7%)
Deaths	234,731	233,185	-1546 (-0.7%)	232,711	-2020 (-0.9%)
Years of life lived	1,247,237	1,297,376	50,139 (4.0%)	1,312,081	64,844 (5.2%)
Quality-adjusted life years	482,038	500,218	18,180 (3.8%)	505,573	23,534 (4.9%)
Acute healthcare costs	$3,127,021,216	$2,897,929,456	-$229,091,760	$3,021,110,848	-$105,910,368
Chronic healthcare costs	$6,730,789,120	$6,889,586,592	$158,797,472	$7,031,207,648	$300,418,528
SGLT2i costs	$105,448,311	$410,299,658	$304,851,348	$110,517,856	$5,069,546
GLP-1 RA costs	$62,227,612	$64,567,413	$2,339,802	$978,289,104	$916,061,492
Total healthcare costs	$10,025,486,258	$10,262,383,119	236,896,861 (2.4%)	$11,141,125,456	1,115,639,198 (11.1%)
Acute absenteeism costs	$0	$0	$0	$0	$0
Chronic absenteeism costs	$0	$0	$0	$0	$0
Non-participation costs - morbidity	$0	$0	$0	$0	$0
Non-participation costs - mortality	$0	$0	$0	$0	$0
Total productivity costs	$0	$0	$0	$0	$0
Total societal costs	$10,025,486,258	$10,262,383,119	$236,896,861	$11,141,125,456	$1,115,639,198
ICER– years of life lived	.	.	4725	.	17,205
ICER – QALYs	.	.	13,031	.	47,405
SICER – years of life lived	.	.	4725	.	17,205
SICER – QALYs	.	.	13,031	.	47,405

All costs are presented in 2020 Australian dollars. All health economic outcomes have been subject to 5% annual discounting.

Abbreviations: SGLT2i – sodium-glucose co-transporter 2 inhibitor; GLP-1 RA – glucagon-like peptide 1 receptor agonist; ICER – incremental cost-effectiveness ratio; QALY – Quality Adjusted Life Year.

ESM Table 8 – Results from Scenario analyses. Incremental costs and benefits are as compared to the current use condition for each scenario.

Total population						
	SGLT2i use			GLP-1 RA use		
Scenario	QALYs	Total Healthcare costs	ICER	QALYs	Total Healthcare costs	ICER
Base-case	176,446	$4,184,773,888	23,717	200,932	$20,234,887,168	100,705
Differential medication costs	176,446	$1,487,034,368	8428	200,932	$16,801,423,360	83,618
SGLT2i in combination with metformin	176,446	$2,575,642,880	14,597	200,932	$20,226,236,416	100,662
Off-patent costs	176,446	$788,084,416	4466	200,932	$10,072,525,824	50,129
Lower adherence	176,446	$2,487,833,344	14,100	200,932	$16,573,888,512	82,485
U.S. medication cost	176,446	$63,002,533,888	357,064	200,932	$124,912,500,736	621,667
Diabetes incidence decreases 4% per year	171,746	$3,807,701,504	22,171	195,332	$18,807,320,576	96,284
Diabetes incidence increases 4% per year	182,449	$4,736,184,320	25,959	208,129	$22,302,822,400	107,159
Decreasing mortality	171,676	$4,019,505,152	23,413	194,877	$20,537,034,752	105,385
GLP-1 RAs reduce ESKD by 22%	176,390	$4,206,257,408	23,846	205,830	$18,782,986,240	91,255
50% use of medications	95,122	$2,252,259,328	23,678	127,866	$12,935,984,128	101,169
100% use of medications	259,719	$6,170,631,168	23,759	275,525	$27,617,202,176	100,235
Private hospitals included	179,539	$3,681,371,136	20,505	202,573	$19,979,929,600	98,631
Timeframe 2020-2030	71,481	$2,939,524,352	41,123	84,266	$12,246,132,736	145,328

Decreasing efficacy 5% per year	124,409	$5,026,678,784	40,404	141,715	$20,163,133,440	142,279
Discounting rate: 0%	323,840	$6,265,424,896	19,347	365,791	$32,547,100,672	88,977
Discounting rate: 3%	222,477	$4,855,525,888	21,825	252,504	$24,154,359,808	95,659
Discounting rate: 6%	157,982	$3,907,565,568	24,734	180,212	$18,634,104,832	103,401
**Secondary prevention population**						
	SGLT2i use			GLP-1 RA use		
Scenario	QALYs	Total Healthcare costs	ICER	QALYs	Total Healthcare costs	ICER
Base-case	29,357	$260,645,232	8878	36,090	$2,877,863,424	79,742
Differential medication costs	29,357	-$95,138,384	Dominant	36,090	$2,424,326,400	67,175
SGLT2i in combination with metformin	29,357	$48,562,112	1654	36,090	$2,876,063,232	79,692
Off-patent costs	29,357	-$187,696,768	Dominant	36,090	$1,537,367,424	42,598
Lower adherence	29,357	$36,751,580	1252	36,090	$2,394,754,560	66,355
U.S. medication cost	29,357	$8,019,593,728	273,174	36,090	$16,695,443,456	462,607
Diabetes incidence decreases 4% per year	28,505	$235,342,096	8256	34,996	$2,736,804,864	78,203
Diabetes incidence increases 4% per year	30,502	$296,715,744	9728	37,567	$3,076,770,048	81,900
Decreasing mortality	29,357	$194,078,224	6611	36,032	$2,987,377,408	82,908
GLP-1 RAs reduce ESKD by 22%	29,346	$265,429,680	9045	37,466	$2,505,744,128	66,880

50% use of medications	15,709	$136,815,264	8709	22,849	$1,831,963,776	80,178
100% use of medications	43,544	$394,097,504	9051	49,749	$3,945,110,528	79,300
Private hospitals included	30,464	$48,148,336	1580	38,689	$3,047,284,992	78,764
Timeframe 2020-2030	11,271	$170,387,744	15,117	14,613	$1,492,741,120	102,154
Decreasing efficacy 5% per year	19,427	$506,764,672	26,085	23,940	$2,810,521,600	117,398
Discounting rate: 0%	54,519	$405,412,544	7436	66,234	$4,914,060,288	74,193
Discounting rate: 3%	37,193	$306,779,360	8248	45,500	$3,520,147,968	77,366
Discounting rate: 6%	26,222	$241,767,856	9220	32,317	$2,617,786,112	81,004

All costs are presented in 2020 Australian dollars. All health economic outcomes have been subject to 5% annual discounting unless otherwise indicated. Abbreviations: SGLT2i – sodium-glucose co-transporter 2 inhibitor; GLP-1 RA – glucagon-like peptide 1 receptor agonist; ICER – incremental cost-effectiveness ratio; QALY – Quality Adjusted Life Year.

ESM Fig. 1 – Age-specific diabetes incidence rates used in the model.

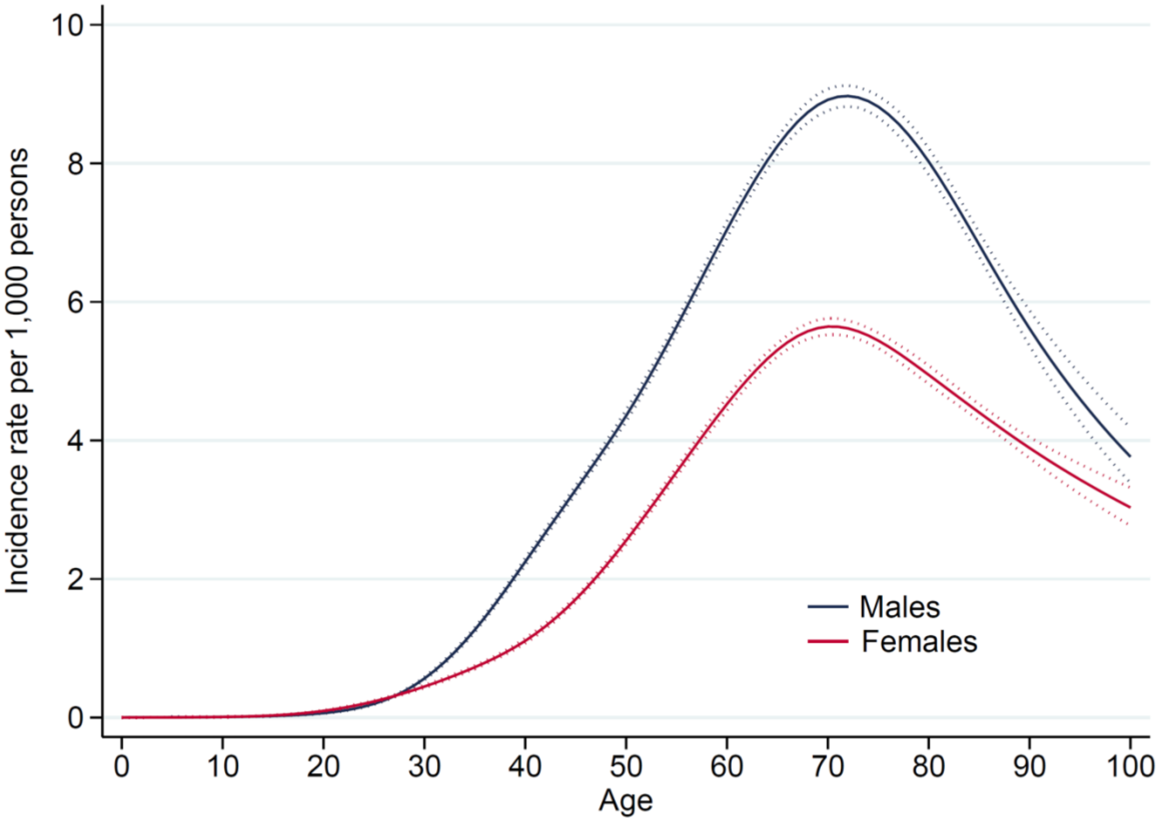

ESM Fig. 2 – Example of the incidence and mortality rates used in the model. Curves show the incidence of hospitalisation for myocardial infarction (MI; **a**), stroke (**b**), and heart failure (HF; **c**), the incidence of end-stage kidney disease (**d**), and all-cause mortality (**e**) by attained age, stratified by sex and age at diagnosis of diabetes (ages 30, 40, 50, 60, 70, and 80). 95% CIs are represented by dotted lines.

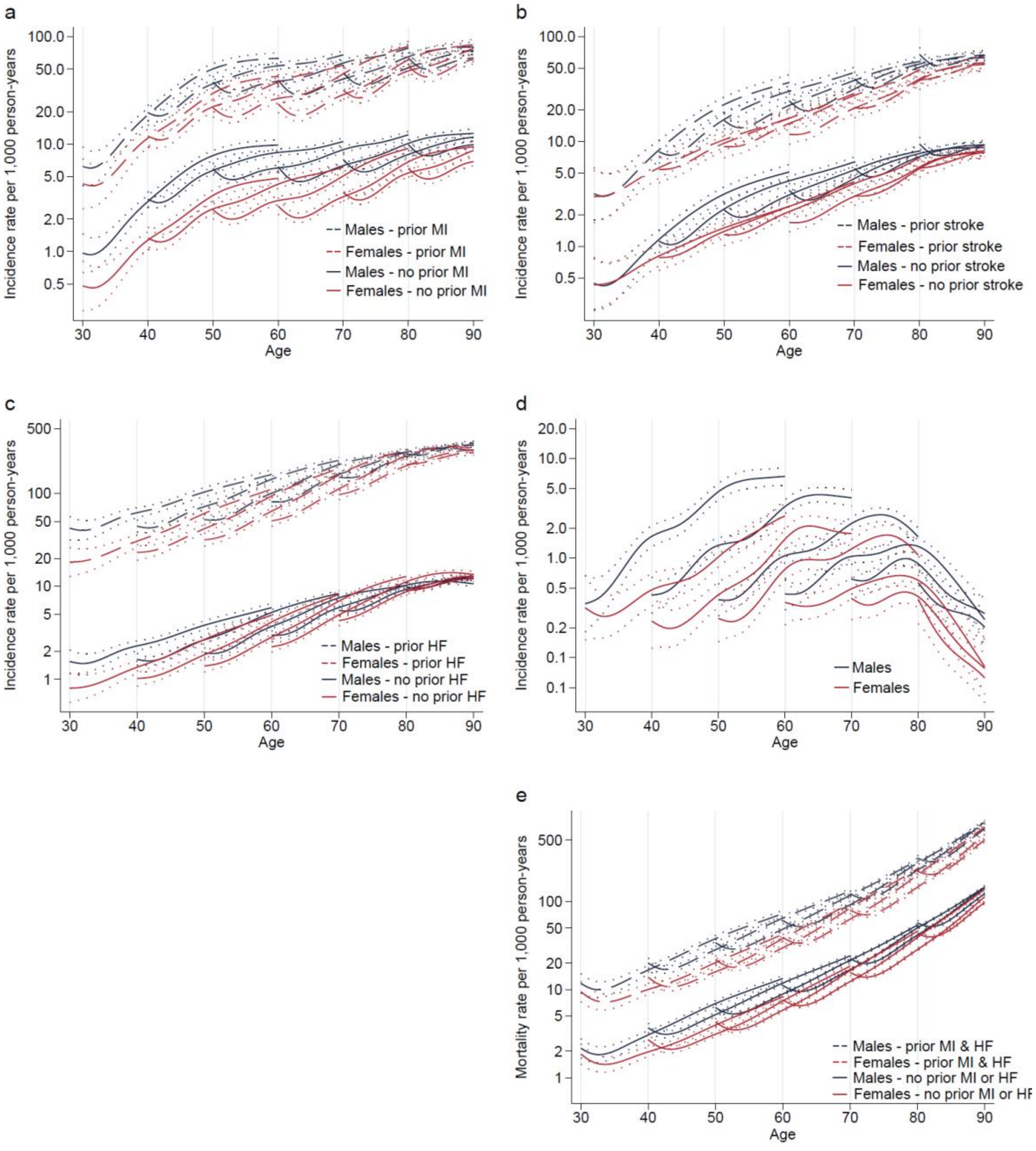

ESM Fig. 3 – Example of the incidence and mortality rates used in the model. Curves show the incidence of hospitalisation for myocardial infarction (MI; **a**), stroke (**b**), and heart failure (HF; **c**), the incidence of end-stage kidney disease (**d**), and all-cause mortality (**e**) by health state for a male aged 60 at diagnosis of diabetes, followed to age 90. Dashed lines indicate a health state for which the outcome is a repeat event.

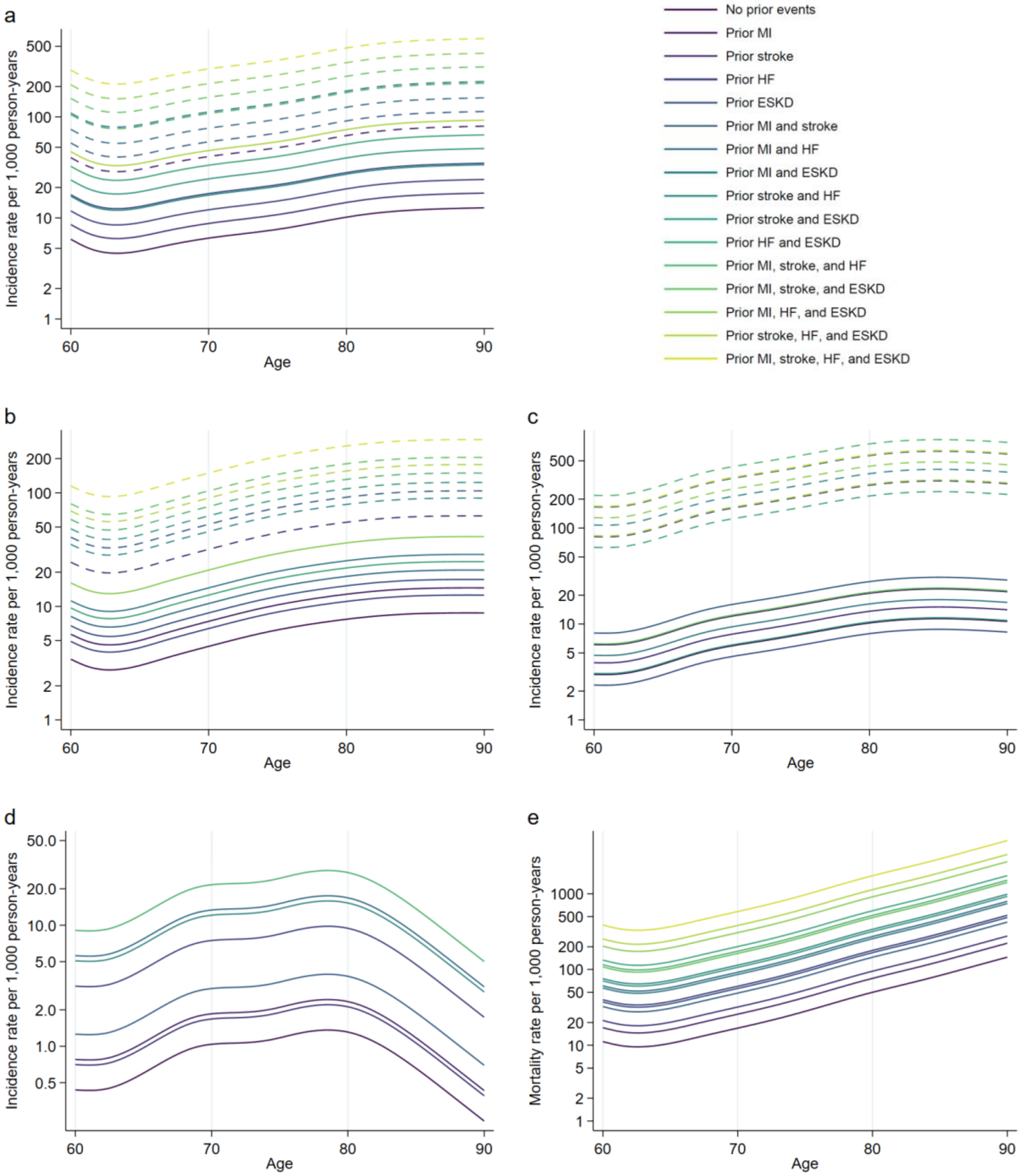

ESM Fig. 4 – Model validation test 1 – Myocardial infarction (MI). Figures show the actual vs. modelled number of MIs, overall (**a**), by sex (**b**), prior MI status (**c**) and age (**d**). See ESM Methods above for more details.

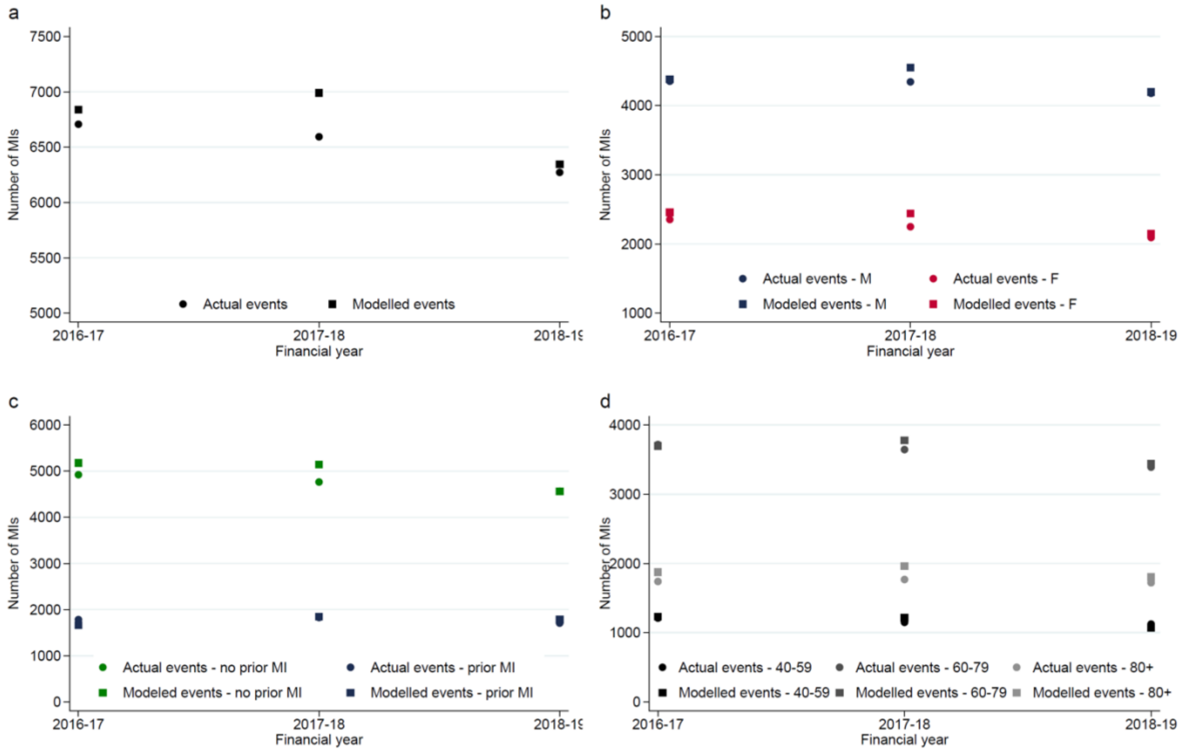

ESM Fig. 5 – Model validation test 1 – Stroke. Figures show the actual vs. modelled number of strokes, overall (**a**), by sex (**b**), prior stroke status (**c**) and age (**d**). See ESM Methods above for more details.

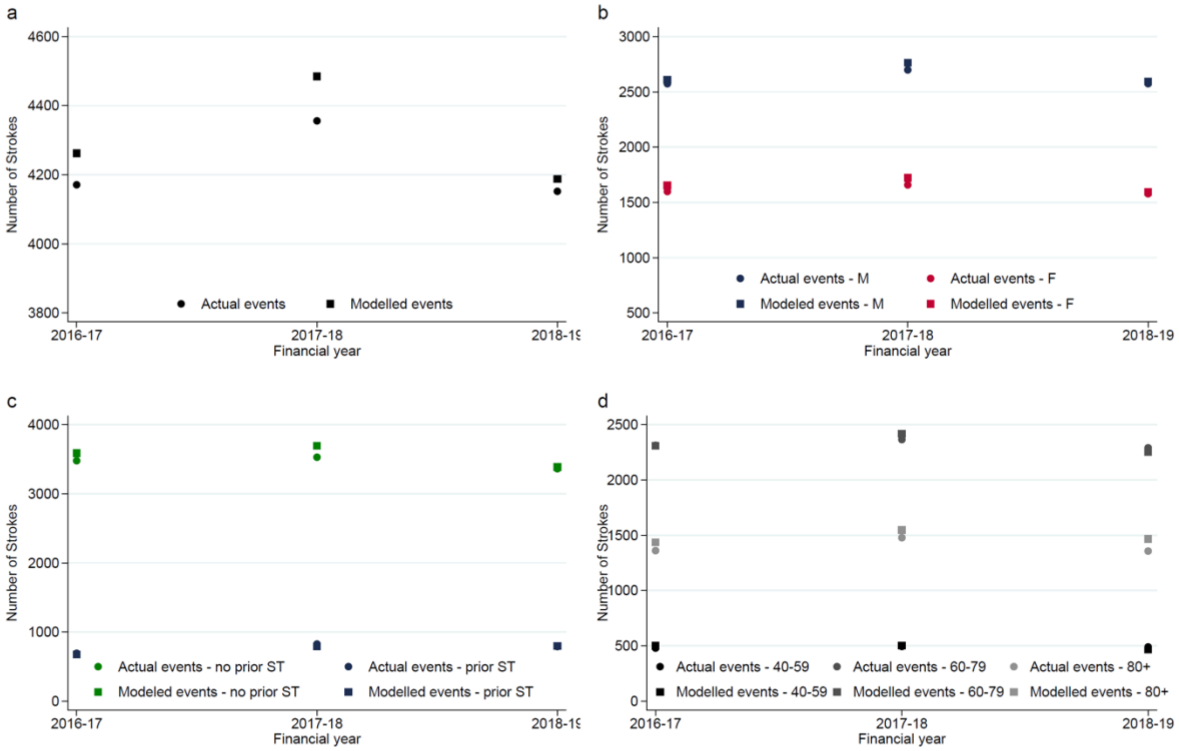

ESM Fig. 6 – Model validation test 1 – Heart failure (HF). Figures show the actual vs. modelled number of HF events, overall (**a**), by sex (**b**), prior HF status (**c**) and age (**d**). See ESM Methods above for more details.

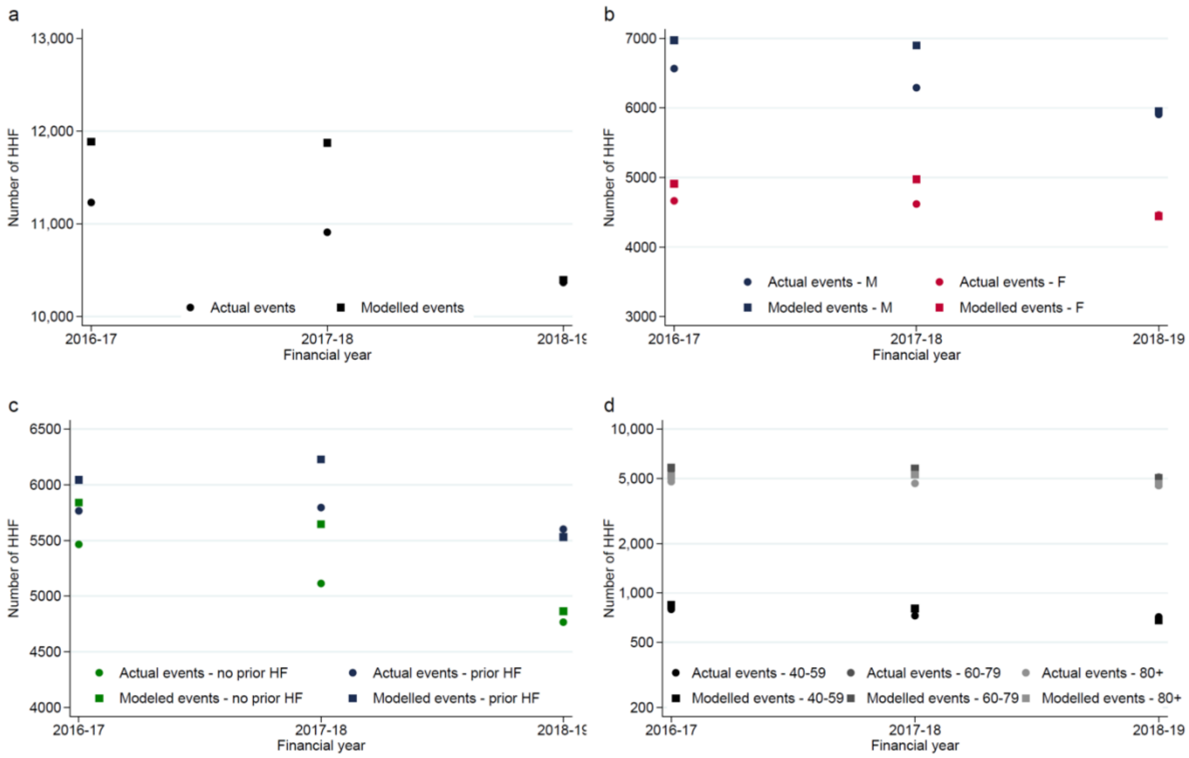

ESM Fig. 7 – Model validation test 1 – End-stage kidney disease (ESKD). Figures show the actual vs. modelled number of ESKD events, overall (**a**), by sex (**b**), and age (**c**). See ESM Methods above for more details.

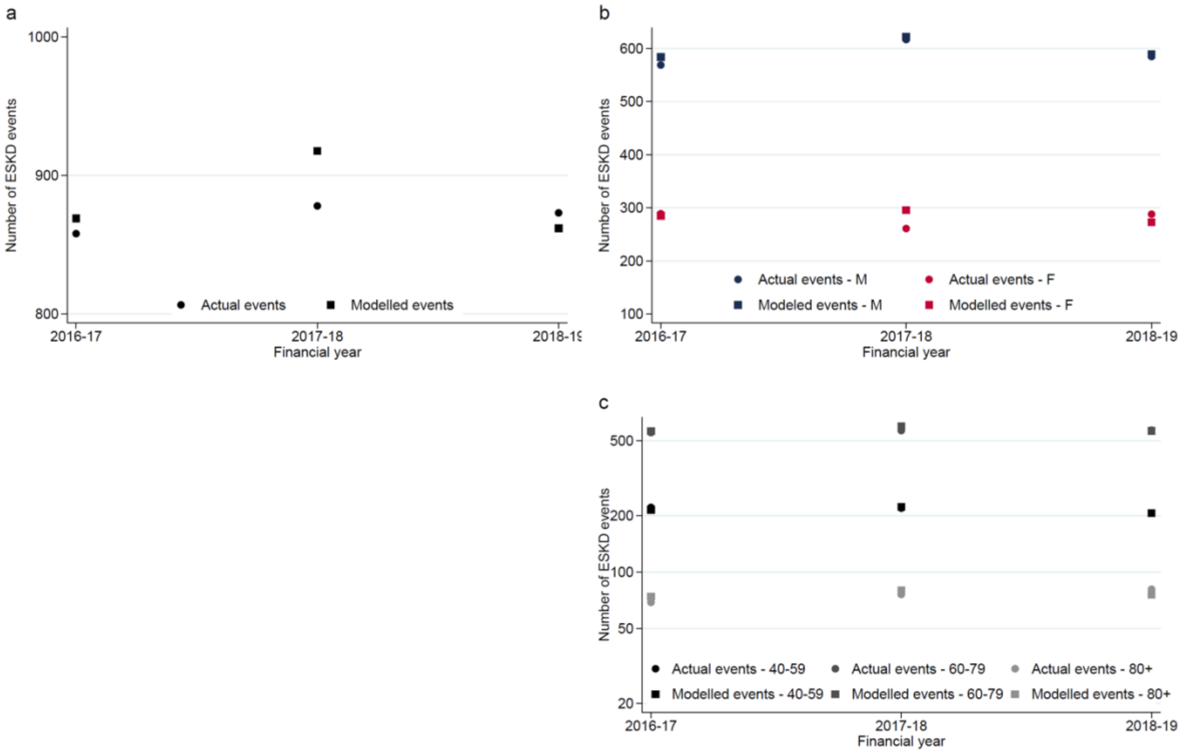

ESM Fig. 8 – Model validation test 1 – All-cause mortality. Figures show the actual vs. modelled number of deaths, overall (**a**), by sex (**b**), and age (**c**). See ESM Methods above for more details.

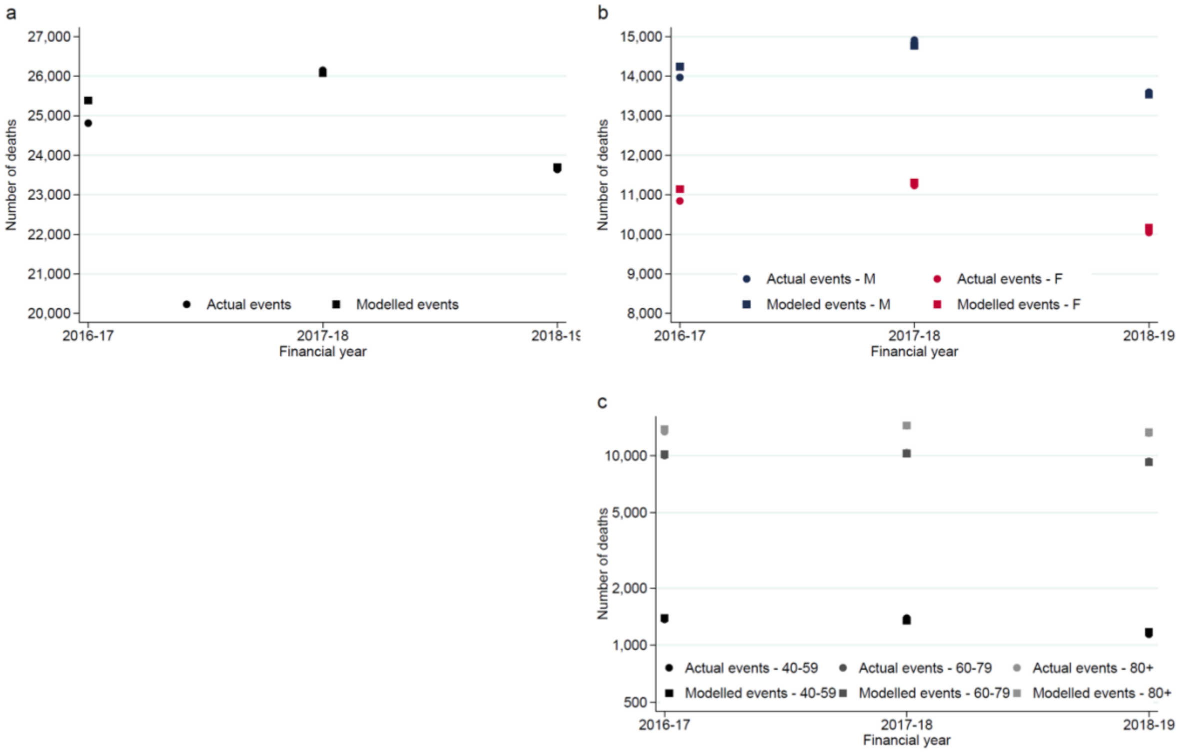

ESM Fig. 9 – Model validation test 2. Figures show the actual vs. modelled number myocardial infarctions (MIs; **a**) strokes (**b**), heart failure (HF) events (**c**), end-stage kidney disease (ESKD) events (**d**), and all-cause mortality events (**e**). See ESM Methods above for more details.

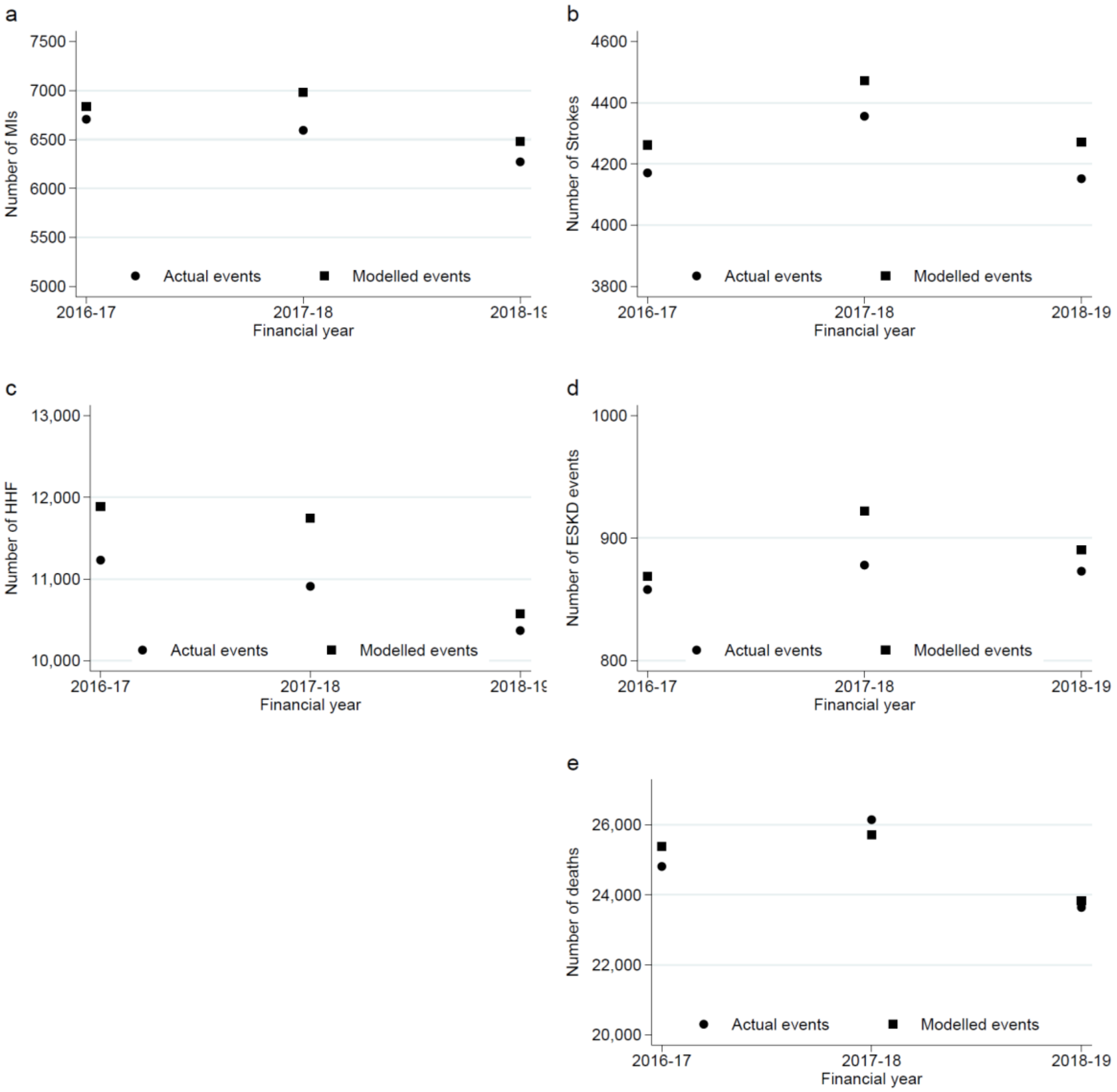

CHEERS 2022 Checklist

Topic	No.	Item	Location where item is reported
**Title**			
	1	Identify the study as an economic evaluation and specify the interventions being compared.	1
**Abstract**			
	2	Provide a structured summary that highlights context, key methods, results, and alternative analyses.	2
**Introduction**			
**Background and objectives**	3	Give the context for the study, the study question, and its practical relevance for decision making in policy or practice.	3
**Methods**			
**Health economic analysis plan**	4	Indicate whether a health economic analysis plan was developed and where available.	N/A
**Study population**	5	Describe characteristics of the study population (such as age range, demographics, socioeconomic, or clinical characteristics).	Appendix - ESM Table 1
**Setting and location**	6	Provide relevant contextual information that may influence findings.	5, 6
**Comparators**	7	Describe the interventions or strategies being compared and why chosen.	3, 7
**Perspective**	8	State the perspective(s) adopted by the study and why chosen.	4, 5
**Time horizon**	9	State the time horizon for the study and why appropriate.	5
**Discount rate**	10	Report the discount rate(s) and reason chosen.	5
**Selection of outcomes**	11	Describe what outcomes were used as the measure(s) of benefit(s) and harm(s).	6
**Measurement of outcomes**	12	Describe how outcomes used to capture benefit(s) and harm(s) were measured.	6
**Valuation of outcomes**	13	Describe the population and methods used to measure and value outcomes.	5, 6, Appendix
**Measurement and valuation of resources and costs**	14	Describe how costs were valued.	Table 1
**Currency, price date, and conversion**	15	Report the dates of the estimated resource quantities and unit costs, plus the currency and year of conversion.	Table 1
**Rationale and description of model**	16	If modelling is used, describe in detail and why used. Report if the model is publicly available and where it can be accessed.	5

Topic	No.	Item	Location where item is reported
**Analytics and assumptions**	17	Describe any methods for analysing or statistically transforming data, any extrapolation methods, and approaches for validating any model used.	Appendix
**Characterising heterogeneity**	18	Describe any methods used for estimating how the results of the study vary for subgroups.	10
**Characterising distributional effects**	19	Describe how impacts are distributed across different individuals or adjustments made to reflect priority populations.	N/A
**Characterising uncertainty**	20	Describe methods to characterise any sources of uncertainty in the analysis.	7, 8
**Approach to engagement with patients and others affected by the study**	21	Describe any approaches to engage patients or service recipients, the general public, communities, or stakeholders (such as clinicians or payers) in the design of the study.	N/A
**Results**			
**Study parameters**	22	Report all analytic inputs (such as values, ranges, references) including uncertainty or distributional assumptions.	Table 1
**Summary of main results**	23	Report the mean values for the main categories of costs and outcomes of interest and summarise them in the most appropriate overall measure.	Table 2
**Effect of uncertainty**	24	Describe how uncertainty about analytic judgments, inputs, or projections affect findings. Report the effect of choice of discount rate and time horizon, if applicable.	Table 2, Fig. 2-4
**Effect of engagement with patients and others affected by the study**	25	Report on any difference patient/service recipient, general public, community, or stakeholder involvement made to the approach or findings of the study	N/A
**Discussion**			
**Study findings, limitations, generalisability, and current knowledge**	26	Report key findings, limitations, ethical or equity considerations not captured, and how these could affect patients, policy, or practice.	12-15
**Other relevant information**			
**Source of funding**	27	Describe how the study was funded and any role of the funder in the identification, design, conduct, and reporting of the analysis	16
**Conflicts of interest**	28	Report authors conflicts of interest according to journal or International Committee of Medical Journal Editors requirements.	16

From: Husereau D, Drummond M, Augustovski F, et al. Consolidated Health Economic Evaluation Reporting Standards 2022 (CHEERS 2022) Explanation and Elaboration: A Report of the ISPOR CHEERS II Good Practices Task Force. Value Health 2022;25. doi:10.1016/j.jval.2021.10.008
